# The discovery of hidden guanylate cyclases (GCs) in the *Homo sapiens* proteome

**DOI:** 10.1016/j.csbj.2023.11.005

**Published:** 2023-11-04

**Authors:** Ilona Turek, Lubna Freihat, Jignesh Vyas, Janet Wheeler, Victor Muleya, David T. Manallack, Chris Gehring, Helen Irving

**Affiliations:** a La Trobe Institute for Molecular Science, La Trobe University, Bendigo, VIC 3550, Australia; b Department of Rural Clinical Sciences, La Trobe Rural Health School, La Trobe University, Bendigo, VIC 3550, Australia; c Drug Discovery Biology, Monash Institute of Pharmaceutical Sciences, Monash University, Parkville, VIC 3052, Australia; d Department of Animal, Plant and Soil Science, La Trobe University, AgriBio building, Bundoora, VIC 3086, Australia; e Medicinal Chemistry, Monash Institute of Pharmaceutical Sciences, Monash University, Parkville, VIC 3052, Australia; f Department of Chemistry, Biochemistry and Biotechnology, University of Perugia, 06121 Perugia, Italy

**Keywords:** Crypto guanylate cyclase, Cyclic GMP (cGMP), Neurotropic receptor tyrosine kinase 1 (NTRK1), Phytosulfokine receptor 1 (PSKR1), Tyrosine receptor kinase A (TrkA)

## Abstract

Recent discoveries have established functional guanylate cyclase (GC) catalytic centers with low activity within kinase domains in plants. These crypto GCs generate guanosine 3’,5’-cyclic monophosphate (cGMP) essential for both intramolecular and downstream signaling. Here, we have set out to search for such crypto GCs moonlighting in kinases in the *H. sapiens* proteome and identified 18 candidates, including the neurotropic receptor tyrosine kinase 1 (NTRK1). NTRK1 shows a domain architecture much like plant receptor kinases such as the phytosulfokine receptor, where a functional GC essential for downstream signaling is embedded within a kinase domain. In vitro characterization of the NTRK1 shows that the embedded NTRK1 GC is functional with a marked preference for Mn^2+^ over Mg^2+^. This therefore points to hitherto unsuspected roles of cGMP in intramolecular and downstream signaling of NTRK1 and the role of cGMP in NTRK1-dependent growth and neoplasia.

## Highlights

• 3',5'-cyclic guanosine monophosphate (cGMP) is a universal second messenger.
• Guanylate cyclases (GCs) catalyze the formation of cGMP from GTP.
• Generation of the GC diagnostic motif led to the discovery of novel GCs in plants.
• The motif query of the human proteome identified proteins that harbor a GC center.
• Several of the candidate GCs identified, including NTRK1, have GC activity.

## Graphical Abstract

**Figure:**
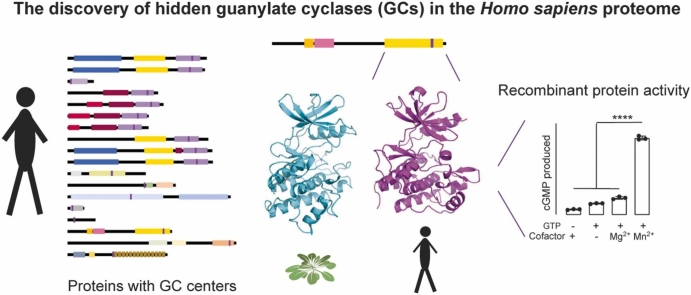

## 1 Introduction

Guanylate cyclases (GCs) catalyze the formation of guanosine 3’,5’-cyclic monophosphate (cGMP) from guanosine-5’-triphosphate (GTP). They fall into two main classes, the soluble GCs and membrane-bound GCs that include the natriuretic peptide receptors [1], [2], [3]. More recently, a third class of moonlighting or crypto GCs has been characterized predominantly in plants. In this new class a GC center is embedded in the kinase domain of the protein [4], [5]. The best studied of these crypto GCs is the plant phytosulfokine receptor 1 (PSKR1) [4], [5], [6]. In animals, the immune checkpoint protein interleukin-1 receptor associated kinase 3 (IRAK3) also contains a functional GC center hidden in a pseudokinase domain [7], [8], [9], [10].

Here we set out to query the *H. Sapiens* proteome with an amino acid motif based on conserved residues essential for catalysis in GCs. This motif has already proven a most useful tool for the identification of functional crypto GCs in plants. The amino acid motif query of the human proteome resulted in 18 proteins predicted to harbor a putative GC center, and most of them have already been characterized as active or putative GCs. We singled out the neurotropic receptor tyrosine kinase 1 (NTRK1) to investigate if our predicted GC was indeed functional. Given that NTRK1 is conserved in vertebrates (mammals, fish, and birds) and essential for the development of nerve growth factor (NGF)-dependent nerve fibers that innervate all tissues of the body [11], [12], [13], we therefore hypothesize that cGMP plays a critical role in this development. Finally, we propose the presence of a number of hitherto undiscovered functional crypto GCs in the *H. sapiens* proteome and that their biological functions will point to new roles for cGMP.

## 2 Materials and methods

### 2.1 Bioinformatic tools

Pattern matching searches in the *Homo sapiens* proteins annotated in the Swiss-Prot database were conducted through ScanProsite (Expasy) [14] (https://prosite.expasy.org/scanprosite/; July 2023) using the GC diagnostic motif [RKS]-[YFW]-[GCTH]-[VIL]-[FV]-X(3)-[VIL]-X(4)-[KR] previously described [15]. Functional enrichments in the network of identified proteins, with the exception of the putative uncharacterized protein MSD5 being a product of a dubious gene prediction (Table 1), were performed against the whole genome in STRING 11.5 database (https://string-db.org/) [16], and domain predictions were done using InterPro 95.0 (https://www.ebi.ac.uk/interpro/) [17]. NTRK1 was used as the search term in the National Center for Biotechnology Information (NCBI) protein data base to identify sequences for alignment with COBALT. Orthologs were aligned in CLUSTAL Omega [18] before creating sequence logos using WebLogo 3 [19]. The PSKR1 (NP_178330.1) protein sequence was used for a non-redundant BLASTp search (maximum of 500) to select orthologs for CLUSTAL Omega alignment and sequence logo creation with WebLogo 3.

**Table 1:** List of human proteins identified in the pattern matching search as putative GCs with the amino acid sequence of the predicted GC catalytic center.

Protein name	UniProtKB ID	Predicted GC center
Atrial natriuretic peptide receptor 1 (ANPR-A)*	P16066	RYCLFgdtVntasR
Atrial natriuretic peptide receptor 2 (ANPR-B)*	P20594	RYCLFgdtVntasR
ADP-ribosylation factor-like protein 4 A (ARL4A)*	P40617	SFHIVilgLdcagK
Guanylate cyclase soluble subunit alpha-1 (GCYA1)*	Q02108	RYCLFgnnVtlanK
Guanylate cyclase soluble subunit alpha-2 (GCYA2)*	P33402	RYCLFgnnVtlasK
Guanylate cyclase soluble subunit beta-1 (GCYB1)*	Q02153	RYCLFgntVnltsR
Guanylate cyclase soluble subunit beta-2 (GCYB2)*	O75343	RYCLFgdtVntasR
Guanylyl cyclase C (GUC2C), intestinal*	P25092	RYCLFgdtVntasR
Guanylyl cyclase 2D (GUC2D), retinal*	Q02846	RYCLFgdtVntasR
Guanylyl cyclase 2 F (GUC2F), retinal*	P51841	RYCLFgdtVntasR
Interleukin-1 receptor-associated kinase 3 (IRAK3)	Q9Y616	SFGIVimeVltgcR
C-Jun-amino-terminal kinase-interacting protein 2 (JIP2)	Q13387	SFGLFsclVngeeR
Methyl methanesulfonate-sensitivity protein 22-like (MMS22)	Q6ZRQ5	SYTIFlciLakvvK
Mitochondrial pyruvate carrier 2 (MPC2)	O95563	KWGLVcagLadmaR
Putative uncharacterized protein (MSD5)∼	A0A3B3IT52	KYHIVskaIaqrlK
Neurotrophic tyrosine receptor kinase 1 (NTRK1)	P04629	SFGVVlweIftygK
X-linked retinitis pigmentosa GTPase regulator-interacting protein 1 (RPGRIP1)	Q96KN7	KFTVVsdpLdeekK
Zinc finger protein 287 (ZN287)	Q9HBT7	SYGIVhrkIlpgeK

* - known to bind GTP; ∼ - product of a dubious gene prediction

### 2.2 Homology model generation

The crystal structure of the TrkA kinase domain [PDB: 4F0I] [20] was used to prepare the homology model of apo-NTRK1. Generation of the PSKR1 kinase domain monomer homology model based on its 41.2 % identity to the tomato Pto (for *Pseudomonas syringae* pv tomato) kinase [PDB: 3HGK] as described previously [7], [21]. Both models were prepared using Prime version 3.1 (Maestro version 9.3, Schrödinger, LLC, New York, USA). Models were minimized using Macromodel version 9.9 (Maestro version 9.3, Schrödinger), employing the PRCG method and the OPLS_2005 force field. Any strain was further reduced by a short period of molecular dynamics (300 K, 10 ps) using Macromodel version 9.9. A Ramachandran analysis (PrimeX 1.9, Maestro version 9.3, Schrödinger) revealed that backbone dihedral angles fell in the expected regions.

### 2.3 Preparation of recombinant NTRK1 constructs and recombinant protein

The NTRK1 gene in pCMV-SPORT6 (IMAGE ID 5200930, accession number BC062580) was amplified with primer sets (Table S6) using Kapa HiFi DNA polymerase (KapaBiosystems, Wilmington, MA, USA) generating Gateway recombination sites and start codon. PCR products were incorporated into pDONR207 (Invitrogen, Carlsbad, CA, USA) forming cytoplasmic domain clones with stop codons (pENTRY-NTRK1-cds) [22], [23] and confirmed by sequencing. Constructs were recombined into bacterial expression vector pDEST17 (ThermoFisher Scientific) to create pDEST17-NTRK1cds. Prior to use all constructs were reconfirmed by sequencing.

*Escherichia coli* BL21-AI or (DE3)pLysS One Shot chemically competent cells (Thermo Fisher Scientific) were transfected with 10 ng of pDEST17-NTRK1cds and positive transformants were selected from LB agar plates containing 50 μg mL^-1^ carbenicillin, and for (DE3)pLysS cells also 34 μg mL^-1^ chloramphenicol. At OD_600_ of ∼0.4, liquid cultures were induced with either 0.2 % (w/v) L-arabinose (Merck) for AI cells or 1 mM isopropylthio-β-galactoside (IPTG; Thermo Fisher Scientific) for (DE3)pLysS cells, for 6 h at 220 rpm at 24 °C. Cells were harvested by centrifugation and N-terminally 6xHis-tagged NTRK1 purified by Ni-NTA agarose (Qiagen) affinity chromatography in the presence of cOmplete EDTA-free protease inhibitor cocktail (Merck) and 1 mM phenylmethylsulfonyl fluoride (PMSF; Merck) at room temperature under native conditions (protocol 12, QIAExpressionist manual (Qiagen)). Amicon Ultra-15 (3000 NMWL cut-off) centrifugal filter units (Merck) were used for buffer exchange (50 mM Tris-HCl pH 7.0, with 1 mM PMSF and cOmplete EDTA-free protease inhibitor cocktail) and concentration of eluted proteins. Protein concentration was measured using a Nanodrop ND-1000 spectrophotometer (Thermo Fisher Scientific) at λ_280_ and purity of preparations was verified on SDS-PAGE.

### 2.4 Assessing GC activity

GC activity was initially screened using a bacterial-based cyclic nucleotide reporter system incorporating plant OLIGOPEPTIDE TRANSPORTER X (OPTX) promoter that is sensitive to cGMP [24]. Briefly, equal amounts of pOPTXcGMPRELUC (#68503 Addgene) and either pDEST17-NTRK1cds or pDESTPSKR1cds [21], [22] were co-transformed into BL21-AI chemically competent cells and grown on LB plates containing selection antibiotics (200 μg mL^-1^ carbenicillin and 100 μg mL^-1^ kanamycin). Single isolated colonies were selected and cultured overnight in Luria-Bertani (LB) broth before diluting culture 1 in 20 into super optimal broth (SOB) (both containing selection antibiotics) and growing until OD_600_ is 0.4 – 0.5. Two 5 mL aliquots were transferred to separate 50 mL tubes with an aliquot induced with 0.2 % L-arabinose for 3 h. After 3 h, a 90 μL aliquot was removed, mixed with 10 μL each of 1 M K_2_HPO_4_ (pH 7.8) and 200 mM EDTA and snap frozen. A further aliquot was used to measure OD_600_ and stored for SDS-PAGE protein analysis. Technical triplicates were assessed for luciferase activity using Promega Luciferase assay system in a LUMIstar Omega microplate reader (BMG Labtech) and expressed relative to OD_600_ values for respective samples and analyzed by one sample Wilcoxon signed rank test with *P* < 0.05 being considered significant.

GC activity of purified 6xHis-tagged NTRK1 protein was measured in vitro by incubating 1 μg protein in 50 mM Tris-HCl, pH 8.0, with 1 mM PMSF and cOmplete EDTA-free protease inhibitor cocktail, 5 mM MgCl_2_ or MnCl_2_ and 1 mM GTP, in a final volume of 100 μL. Background cGMP levels were measured in incubation medium with no protein as well as mixtures containing combinations of the reaction components minus one ingredient (negative controls). Reactions were incubated for 20 min at room temperature (20 °C) and terminated by boiling for 3 min, cooling the tubes on ice for 2 min, followed by centrifugation at 2300× *g* for 3 min. Resulting clarified supernatant was assayed for cGMP content using cGMP EIA Biotrak System (GE Healthcare) following the acetylation protocol according to the manufacturer’s recommendations. Spectrophotometric measurements were performed at λ_405_ using the CLARIOstar Plus (BMG Labtech) and data from three independent experiments were analyzed using one-way ANOVA followed by Tukey-Kramer multiple comparison test with *P* < 0.05 being considered significant.

## 3 Results

### 3.1 Identification of hidden GC catalytic centers in human proteins

**Fig. 1:**
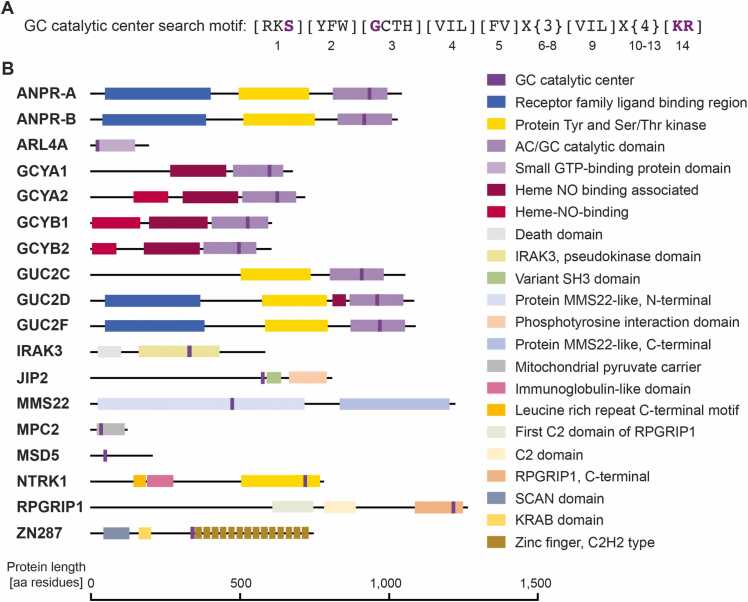
Identification of putative GCs in the human proteome. A. The search motif used to retrieve NTRK1 with functionally assigned amino acids shown in purple. B. Domain organization of the identified proteins with the putative GC catalytic center indicated. GC – guanylate cyclase, AC – adenylate cyclase, GTP – guanosine-5’-triphosphate, NO – nitric oxide, SH3 – Src homology 3, MMS22 – methyl methanesulfonate-sensitivity protein 22-like, RPGRIP1 – retinitis pigmentosa G-protein regulator interacting protein 1, SCAN – SRE-ZBP, CTfin51, AW-1 and Number 18 cDNA, KRAB – Krüppel-associated box.

Since we had identified functional GCs in the model plant *Arabidopsis thaliana* using amino acid pattern matching searches in the *Arabidopsis thaliana* proteome and in the TAIR database [25], we were keen to see if searches of proteomes of other species would reveal further crypto GC centers. Hence, we carried out searches using the GC diagnostic motif [RKS]-[YFW]-[GCTH]-[VIL]-[FV]-X(3)-[VIL]-X(4)-[KR] in ScanProsite [14]. The residue in position 1 forms a hydrogen bond with the guanine moiety of the GTP substrate, while the substrate specificity is conferred by residue in position 3. The positively-charged residue in position 14 stabilizes the transition from GTP to the cGMP product [15] (Fig. 1A). This motif was identified in a total of 18 human proteins pointing to the putative GC catalytic centers (Table 1, Table S1). It is noteworthy that the 18 proteins include several well-characterized soluble GCs (e.g., GCYA1, GCYB1). Gene Ontology (GO) analyses of the identified proteins showed significant enrichment of terms related to GC signaling and activity (Table S2). The significantly enriched biological processes GO categories include ‘receptor GC signaling pathway’, ‘cGMP-mediated signaling’, ‘positive regulation of cGMP-mediated signaling’ and in the molecular function category the significantly enriched terms include ‘GC activity’ and ‘GTP binding’, while in the cellular component category the enriched terms included ‘GC complex, soluble’ (Table S2).

Many of the identified proteins are annotated as receptors with a kinase domain (Fig. 1B). The functional enrichment of the domains of the proteins identified as putative GCs were observed for domains and features including adenylate and guanylate cyclase catalytic domain, receptor family ligand binding region, protein kinase domain, and heme NO binding associated domain (Table S3). Furthermore, pathways such as purine metabolism, cGMP-protein kinase G (PKG) signaling, and NO/cGMP/PKG mediated neuroprotection are significantly enriched for the set of the putative GCs identified (Table S4). The STRING cluster of the identified proteins showed enrichment in terms including cGMP metabolic process and cGMP-dependent kinase (or PKG), natriuretic peptide, and adenylyl cyclase class-4/guanylyl cyclase (Table S5).

Among the identified GCs we noted well-characterized GCs such as atrial natriuretic peptide receptor 1 and 2 (ANPR-A, ANPR-B) and soluble GCs (e.g., GCYA1, GCYB1) further increasing confidence in the validity of the search motif. The list also includes interleukin-1 receptor-associated kinase 3 (IRAK3), but not other members of the IRAK family (Table 1). Similarly, among the identified putative GCs we noted NTRK1, but not NTRK2 or NTRK3 (Table 1). While IRAK3 has been confirmed as a human crypto GC [7], [8], [10], we set out to investigate the structural and functional features NTRK1 with its predicted GC catalytic center embedded in the kinase (Fig. 1).

### 3.2 The domain architecture of NTRK1

**Fig. 2:**
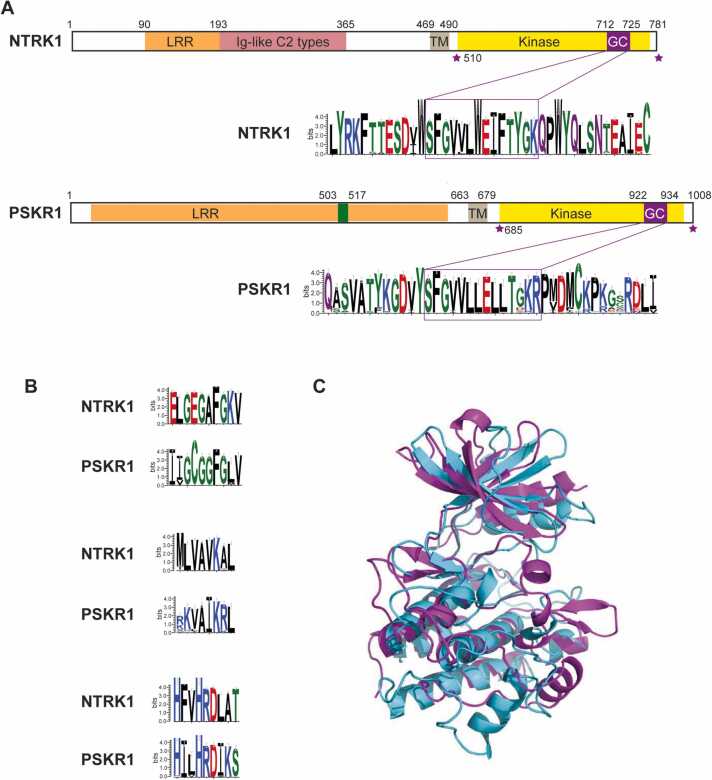
Domain structure of NTRK1 and PSKR1. A. Similarities in the domain architecture of NTRK1 and PSKR1 (sequence numbering is for the human NTRK1 and Arabidopsis PSKR1). The sequence logo of the area surrounding the GC center is shown for 217 NTRK1 orthologs and 98 orthologs of PSKR1. Full Clustal Omega alignment of the GC regions is shown in Fig. S2. LRR – leucine rich repeat, TM – transmembrane, GC - GC center, area between purple asterisks indicates the cloned cytoplasmic domain. B. Sequence logos of NTRK1 and PSKR1 orthologs comparing the kinase G rich, VAIK and HRD regions used to pinpoint the overlay of the homology models. **C**. Superposition of NTRK1 (magenta) and PSKR1 (cyan) overlaid against G-loop, VAIK, HRD, V657(NTRK1) against L869(PSKR1) motifs. Pairwise Clustal Omega alignment of the kinase domains of NTRK1 and PSKR1 is shown in Fig. S1.

To determine if a GC center is also present in NTRK1 orthologs we searched the NCBI protein database using NTRK1 as the search term and identified 238 orthologs from Tetrapoda. Sequence alignments revealed several sequences with large inserts or deletions in the kinase homology domain. These sequences were removed and the remaining 217 orthologous sequences were aligned in CLUSTAL Omega [18] before creating sequence logos using WebLogo 3 [19] (Fig. 2A; Fig. S2A). A non-redundant BLASTp search revealed 98 PSKR1 orthologs from Eudicota that were used for CLUSTAL Omega alignment and sequence logo creation (Fig. 2A; Fig. S2B).

Similarity between the domain architecture of NTRK1 and plant receptor crypto GCs is apparent and highlighted in PSKR1 as an example (Fig. 2A and Fig. S1). Both proteins are membrane-bound receptor kinases that dimerize upon ligand binding [13], [26], [27]. Notably the GC center is found embedded in the same region of the kinase domain and contains the highly conserved serine, glycine and basic residues at the appropriate positions (Fig. 2A and Fig. S1). These amino acids are marked in purple in the search motif (Fig. 1A) and are functionally assigned residues in the GC catalytic center. The serine residue in position 1 has the capacity to form a hydrogen bond with the guanine moiety of GTP. The glycine residue in position 3 confers substrate specificity for GTP. The basic residue at position 14 (lysine for NTRK1 and arginine for PSKR1) has a role in stabilizing the transition from GTP to cGMP [15].

To further examine similarities of the GC regions in NTRK1 and PSKR1, we generated homology models of the kinase domains of these proteins. We used the G-loop, VAIK and HRD motifs [28], [29] that are typically present in kinases and are points of reference to overlay these models to assess and confirm the overall similarity of the proteins (Fig. 2B and C). Both molecules show the typical kinase fold as expected as they are active kinases with NTRK1 being a tyrosine kinase [30] and PSKR1 being a dual function serine/threonine and tyrosine kinase [31].

### 3.3 Recombinant NTRK1 generates cGMP

**Fig. 3:**
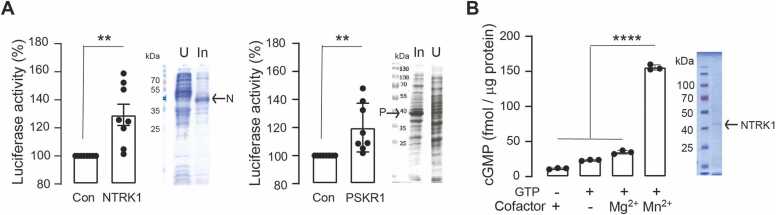
NTRK1 has guanylate cyclase activity. A Detection of cGMP using the cGMP sensitive luciferase reporter system. Bacteria were co-transformed with pOPTXcGMPRELUC and either pDEST17-NTRK1cds or pDEST17-PSKR1cds and induced with arabinose for 3 h before assessing luciferase activity in the bacteria. Control (Con) cultures were not induced and grown under the same conditions. Treatments were normalized to individual transformant controls and analyzed by one sample Wilcoxon signed rank test (** P = 0.0078, n = 8, discrepancy 25.14 (NTRK1) or 12.58 (PSKR1)). Exemplars of total protein in bacteria expressing recombinant NTRK1 cytoplasmic domain (expected 40.1 kDa) or PSKR1 cytoplasmic domain (expected 40 kDa) and separated by SDS-PAGE; U – uninduced, In – induced. **B.** Enzyme-linked immunoassay quantification of cGMP generated in vitro by the cytoplasmic domain of NTRK1 in the presence of cofactors. Data shown as mean ± SD, n = 3; one-way ANOVA followed by Tukey’s post-hoc test (**** P < 0.0001). Exemplar NTRK1 cytoplasmic domain preparation eluted under low imidazole concentrations and separated by SDS-PAGE.

The presence of a GC center motif in a kinase domain is relatively rare. However, given that several plant receptors have functional GC centers embedded in kinases, we tested the functionality of the NTRK1 GC. Firstly, we prepared cytoplasmic domain constructs of NTRK1 containing the kinase domain with embedded GC center (Fig. 2A). Then we co-expressed these constructs with a GC:luciferase reporter system containing a promoter responsive to cGMP fused to a luciferase reporter gene in bacteria to screen for nucleotide cyclase activity [24]. Relative increases in luciferase activity were detected following induction of either NTRK1 or PSKR1 in the transformed bacteria. Induction of NTRK1 or PSKR1 expression in the bacteria is confirmed by the strong bands marked in the crude lysate preparations (Fig. 3A). After normalizing the different bacterial transformants to their uninduced controls, it is evident that induction of NTRK1 or PSKR1 protein expression caused similar raised levels of luciferase activity (Fig. 3A). This observation is reflective of nucleotide cyclase activity by both NTRK1 and PSKR1 in the non-homologous bacterial screening system.

Then we tested if the recombinant NTRK1 cytoplasmic domain has the ability to generate cGMP in vitro using enzyme-linked cGMP immunoassays. NTRK1 recombinants showed a strong preference for Mn^2+^ as the cofactor in these assays where in the presence of Mn^2+^ in 15 min a µg protein generates over 100 fmol of cGMP more than in the presence of Mg^2+^ (Fig. 3B). This is similar to IRAK3, where full-length protein under the same conditions is capable of generating per 1 µg recombinant approximately 300 fmol of cGMP more in the presence of Mn^2+^ than in the presence of Mg^2+^ [8]. While a truncated version of IRAK3 just containing the pseudokinase can generate approximately 1400 fmol more cGMP when Mn^2+^ is present compared to Mg^2+^ [10]. Such cofactor preference is distinct from PSKR1 cytoplasmic domain which can use either Mn^2+^ or Mg^2+^ to generate over 40 fmol cGMP per 1 µg in 15 min [6], [31]. Together these findings (Fig. 3) are evidence NTRK1 has indeed catalytic GC activity.

## 4 Discussion

An increasing number of proteins are being shown to contain hidden nucleotide cylcase motifs conferring catalytic activity essential for biological function. This phenomenon is evident across the kingdoms of life with some notable examples including TIR/AFB auxin receptors [32] and brassinosteroid receptors [23], [25] in plants. Even more remarkably, some proteins like *Arabidopsis thaliana* K^+^-uptake permease contain both the “on” and “off” switches exhibiting dual cyclic nucleotidase and phosphodiesterase activity in separate domains so they have the capacity to both generate cAMP and degrade it [33]. These crypto cyclase activities were initially identified using searches for amino acid motifs diagnostic for nucleotide cyclase activity [15], [33] and it is likely more moonlighting proteins with crypto catalytic activities will be discovered. Here we applied such an amino acid motif-based approach to identify crypto GCs in the *H. sapiens* proteome. The search identified 18 proteins (Table 1), including IRAK3 [7], [8], [10] and NTRK1.

A comparison of NTRK1 with PSKR1, a well-characterized plant receptor kinase containing a crypto GC center [6], [21], [31], shows high homology at the GC center (identity = 72.73 %, coverage = 78 %, E-value = 2e^-6^) and across the kinase domain (identity = 29.29 %, coverage = 71 %, E-value = 9e^-22^). The kinase domains of both NTRK1 and PSKR1 contain the key community maps enabling fine tuning of kinase activity [5], [28]. Both proteins are active kinases [13], [26], [27], [30], [31], so this is not surprising. Both NTRK1 and PSKR1 stimulated a non-homologous screening bacterial system to generate cGMP shown by increases in luciferase activity. The ability of NTRK1 to generate cGMP was further confirmed using recombinant proteins in an ELISA based assay, where cGMP was produced in vitro in the presence of the cofactor Mn^2+^ but not Mg^2+^.

Crypto GCs characterized to-date generate comparatively small amounts of cGMP in vitro, as 1 µg of the crypto GC recombinants can generate approximately 40 – 1500 fmol cGMP in 15 min at room temperature [6], [8], [10], [21] and NTRK1 is no exception. This feature is thought to be, in part, due to the hypothesized role of these crypto GCs to create a narrow cGMP-enriched nanodomain surrounding the protein that enables specific interactions with nearby downstream proteins [5]. In comparison cGMP levels generated by animal soluble GCs under comparable reaction conditions ranging from high femtomolar to nanomolar concentrations [34], [35]. Membrane bound GCs transfected into heterologous cell systems produce picomolar amounts of cGMP [36], [37], while the GC activity of NPR-A and NPR-B receptors studied in membrane preparations isolated from rat hearts showed femtomolar levels of cGMP generated by µg protein per min at the basal level and upon ligand stimulation [38]. Low femtomolar levels of cGMP were also generated by GUCY2D transfected into HeLa cells [39], indicating that the amount of cGMP generated by crypto GCs may be physiologically relevant. Moreover, it is likely that additional ion and protein cofactors may be missing in the in vitro conditions. For instance, PSKR1 GC activity is increased in the presence of Ca^2+^ ions which at the same concentrations inhibit PSKR1 kinase activity [21] providing extra subtle control over cGMP generation. Similarly, activity of recombinant olfactory rodent GC-D (Gucy2D or Gucy2E) is increased by bicarbonate ions [40]. Constraints of protein conformation also probably play a part as a truncated version of IRAK3 containing the pseudokinase domain alone is more active than the full length protein [10] as also observed with GC-A (ANPR-A) [41]. Furthermore, increases in cGMP decrease kinase activity of plant crypto GC kinases [6], [42] indicating an additional layer of intramolecular self-regulation over and above phosphorylation that potentially tunes specific responses to environmental cues.

Underscoring its role in development and regulating cell growth, NTRK1 is also widely studied as tyrosine receptor kinase A (TrkA) which forms fusion proteins occurring in numerous tumor types [26]. The kinase domain of NTRK1 is found fused with potentially many other proteins and these fusions form a diagnostic pattern for some rare cancers and are a relatively low but consistent occurrence for more common cancers [26]. NGF binding stimulates NTRK1 autophosphorylation of tyrosine residues to provide scaffolds for activating mitogen activated protein kinase, phosphatidylinositol 3 kinase and protein kinase C/calcium pathways involved in nerve outgrowth, cell survival, growth and synaptic plasticity [13]. Several earlier reports implicated cGMP as a potential second messenger in NGF mediated signaling. NGF induced cGMP levels and stimulated cGMP phosphodiesterase activity in rat pheochromocytoma PC12 cells [43]. Further, modulating cGMP-protein kinase (PKG) activity switched rat superior cervical ganglion axon guidance responses to NGF gradients [44], while a cGMP analog increased NGF-mediated nerve elongation [45]. NGF is also found at sites of injury and inflammation where it down regulates innate immune responses via activating NTRK1 expressed in monocytes and macrophages [46]. This finding underlines similarities between nerve and immune cells. Fascinatingly as IRAK3, the only known animal crypto GC, is found predominantly in monocytes and macrophages, it raises the possibility that crypto GCs have several roles in these immune cells. We hypothesize that crypto GC of NTRK1 generates a cGMP-enriched nanoenvironment around NTRK1 that could enable interactions between proteins in the signaling cascades; much as proposed for IRAK3, where inactivation of its GC center hinders it’s ability to downregulate immune function [8], [9], [10] pointing to the need of a cGMP-enriched nanodomain surrounding IRAK3. Given the biological roles of NTRK1 and its mode of action, it will be interesting to investigate what the functional role of the GC and hence cGMP is and how this could be harnessed to facilitate development of further therapeuticals in addition to the currently available NTRK1/TrkA tyrosine kinase inhibitors.

## 5 Conclusions

Here we identified a novel crypto catalytic site in NTRK1 and used in vitro approaches to confirm that it has low GC activity that may contribute to highly localized enriched cGMP nanodomains enabling fine tuning of protein behavior [5]. Further investigations using cell studies including genetic ablation are warranted.

## Funding

This research was partly funded by Australian Research Council’s Discovery funding scheme project number DP110104164 and Monash Institute of Pharmaceutical Science Large Grant Support Scheme. LF and VM received scholarships from the Monash Institute of Pharmaceutical Sciences.

## CRediT authorship contribution statement

**Ilona Turek:** Data curation, Formal analysis, Investigation, Methodology, Visualization, Writing - review & editing. **Lubna Freihat:** Methodology, Formal analysis, Investigation. **Jignesh Vyas:** Investigation. **Janet Wheeler:** Methodology, Formal analysis, Investigation, Supervision. **Victor Muleya:** Investigation. **David T. Manallack:** Methodology, Investigation, Writing – review and editing, Visualization, Supervision, Funding acquisition. **Chris Gehring:** Writing – review and editing. **Helen Irving:** Conceptualization, Formal Analysis, Resources, Data curation, Writing – original draft, Writing – review and editing, Visualization, Supervision, Project administration, Funding acquisition.

## Declarations of Competing Interest

None.

## Data Availability

All data are reported in the manuscript and in the Supplementary Material.
