# Supplementary material for: The discovery of hidden guanylate cyclases (GCs) in the Homo sapiens proteome
Source: Comput Struct Biotechnol J. 2023 Nov 4;21:5523–9. doi: 10.1016/j.csbj.2023.11.005 (PMC10665587; doi:10.1016/j.csbj.2023.11.005)
Supplement: Supplementary file 1 — Supplementary material [file mmc1.docx]

**Supplementary Materials for**

The discovery of hidden GCs in the *Homo sapiens* proteome

Ilona Turek, Lubna Freihat, Jignesh Vyas, Janet Wheeler, Victor Muleya, David T. Manallack, Chris Gehring and Helen Irving*

* Correspondence: [h.irving@latrobe.edu.au](mailto:h.irving@latrobe.edu.au)

The following pages include:

**Supplementary Tables**

**Table S1.** Amino acid sequences of human proteins identified as putative GCs in the pattern matching search.

**Table S2.** Gene Ontology (GO) enrichment analysis of the proteins identified as putative GCs.

**Table S3.** Domain enrichment analysis of the proteins identified as putative GCs.

**Table S4.** Pathway enrichment analysis of the proteins identified as putative GCs.

**Table S5.** Functional enrichment analysis in the local network cluster, disease-gene associations, and subcellular localization of the proteins identified as putative GCs.

**Table S6.** List of primers used in cloning NTRK1 constructs.

**Supplementary Figures**

**Figure S1**. Alignment of the kinase domain of NTRK1 and PSKR1 used for homology modelling.

**Figure S2**. Alignment of the guanylate cyclase (GC) center of NTRK1 (A) and PSKR1 (B).

**Table S1.** Amino acid sequences of human proteins identified as putative GCs in the pattern matching search. The ScanProsite search among *Homo sapiens* proteins in the Swiss-Prot database was performed using the motif [RKS][YFW][GCTH][VIL][FV]-X(3)-[VIL]-X(4)-[KR]. Isoforms are included.

| **Protein sequence with the position and amino acid sequence of the predicted GC centre (in blue)** |
| --- |
| **>sp\|P16066\|ANPRA_HUMAN** (1061 aa) Atrial natriuretic peptide receptor 1 (EC 4.6.1.2) (Atrial natriuretic peptide receptor type A) (ANP-A) (ANPR-A) (NPR-A) (Guanylate cyclase A) (GC-A) MPGPRRPAGSRLRLLLLLLLPPLLLLLRGSHAGNLTVAVVLPLANTSYPWSWARVGPAVELALAQVKARPDLLPGWTVRTVLGSSENALGVCSDTAAPLAAVDLKWEHNPAVFLGPGCVYAAAPVGRFTAHWRVPLLTAGAPALGFGVKDEYALTTRAGPSYAKLGDFVAALHRRLGWERQALMLYAYRPGDEEHCFFLVEGLFMRVRDRLNITVDHLEFAEDDLSHYTRLLRTMPRKGRVIYICSSPDAFRTLMLLALEAGLCGEDYVFFHLDIFGQSLQGGQGPAPRRPWERGDGQDVSARQAFQAAKIITYKDPDNPEYLEFLKQLKHLAYEQFNFTMEDGLVNTIPASFHDGLLLYIQAVTETLAHGGTVTDGENITQRMWNRSFQGVTGYLKIDSSGDRETDFSLWDMDPENGAFRVVLNYNGTSQELVAVSGRKLNWPLGYPPPDIPKCGFDNEDPACNQDHLSTLEVLALVGSLSLLGILIVSFFIYRKMQLEKELASELWRVRWEDVEPSSLERHLRSAGSRLTLSGRGSNYGSLLTTEGQFQVFAKTAYYKGNLVAVKRVNRKRIELTRKVLFELKHMRDVQNEHLTRFVGACTDPPNICILTEYCPRGSLQDILENESITLDWMFRYSLTNDIVKGMLFLHNGAICSHGNLKSSNCVVDGRFVLKITDYGLESFRDLDPEQGHTVYAKKLWTAPELLRMASPPVRGSQAGDVYSFGIILQEIALRSGVFHVEGLDLSPKEIIERVTRGEQPPFRPSLALQSHLEELGLLMQRCWAEDPQERPPFQQIRLTLRKFNRENSSNILDNLLSRMEQYANNLEELVEERTQAYLEEKRKAEALLYQILPHSVAEQLKRGETVQAEAFDSVTIYFSDIVGFTALSAESTPMQVVTLLNDLYTCFDAVIDNFDVYKVETIGDAYMVVSGLPVRNGRLHACEVARMALALLDAVRSFRIRHRPQEQLRLRIGIHTGPVCAGVVGLKMPRYCLFGDTVNTASRMESNGEALKIHLSSETKAVLEEFGGFELELRGDVEMKGKGKVRTYWLLGERGSSTRG  **991 - 1004:     RYCLFgdtVntasR** |
| **>sp\|P20594\|ANPRB_HUMAN**(1047 aa) Atrial natriuretic peptide receptor 2 (EC 4.6.1.2) (Atrial natriuretic peptide receptor type B) (ANP-B) (ANPR-B) (NPR-B) (Guanylate cyclase B) (GC-B) MALPSLLLLVAALAGGVRPPGARNLTLAVVLPEHNLSYAWAWPRVGPAVALAVEALGRALPVDLRFVSSELEGACSEYLAPLSAVDLKLYHDPDLLLGPGCVYPAASVARFASHWRLPLLTAGAVASGFSAKNDHYRTLVRTGPSAPKLGEFVVTLHGHFNWTARAALLYLDARTDDRPHYFTIEGVFEALQGSNLSVQHQVYAREPGGPEQATHFIRANGRIVYICGPLEMLHEILLQAQRENLTNGDYVFFYLDVFGESLRAGPTRATGRPWQDNRTREQAQALREAFQTVLVITYREPPNPEYQEFQNRLLIRAREDFGVELGPSLMNLIAGCFYDGILLYAEVLNETIQEGGTREDGLRIVEKMQGRRYHGVTGLVVMDKNNDRETDFVLWAMGDLDSGDFQPAAHYSGAEKQIWWTGRPIPWVKGAPPSDNPPCAFDLDDPSCDKTPLSTLAIVALGTGITFIMFGVSSFLIFRKLMLEKELASMLWRIRWEELQFGNSERYHKGAGSRLTLSLRGSSYGSLMTAHGKYQIFANTGHFKGNVVAIKHVNKKRIELTRQVLFELKHMRDVQFNHLTRFIGACIDPPNICIVTEYCPRGSLQDILENDSINLDWMFRYSLINDLVKGMAFLHNSIISSHGSLKSSNCVVDSRFVLKITDYGLASFRSTAEPDDSHALYAKKLWTAPELLSGNPLPTTGMQKADVYSFGIILQEIALRSGPFYLEGLDLSPKEIVQKVRNGQRPYFRPSIDRTQLNEELVLLMERCWAQDPAERPDFGQIKGFIRRFNKEGGTSILDNLLLRMEQYANNLEKLVEERTQAYLEEKRKAEALLYQILPHSVAEQLKRGETVQAEAFDSVTIYFSDIVGFTALSAESTPMQVVTLLNDLYTCFDAIIDNFDVYKVETIGDAYMVVSGLPGRNGQRHAPEIARMALALLDAVSSFRIRHRPHDQLRLRIGVHTGPVCAGVVGLKMPRYCLFGDTVNTASRMESNGQALKIHVSSTTKDALDELGCFQLELRGDVEMKGKGKMRTYWLLGERKGPPGLL  **976 - 989:      RYCLFgdtVntasR** |
| **>sp\|P40617\|ARL4A_HUMAN** (200 aa) ADP-ribosylation factor-like protein 4A MGNGLSDQTSILSNLPSFQSFHIVILGLDCAGKTTVLYRLQFNEFVNTVPTKGFNTEKIKVTLGNSKTVTFHFWDVGGQEKLRPLWKSYTRCTDGIVFVVDSVDVERMEEAKTELHKITRISENQGVPVLIVANKQDLRNSLSLSEIEKLLAMGELSSSTPWHLQPTCAIIGDGLKEGLEKLHDMIIKRRKMLRQQKKKR **20 - 33:        SFHIVilgLdcagK** |
| **>sp\|Q02108\|GCYA1_HUMAN**(690 aa) Guanylate cyclase soluble subunit alpha-1 (GCS-alpha-1) (EC 4.6.1.2) (Guanylate cyclase soluble subunit alpha-3) (GCS-alpha-3) (Soluble guanylate cyclase large subunit) MFCTKLKDLKITGECPFSLLAPGQVPNESSEEAAGSSESCKATVPICQDIPEKNIQESLPQRKTSRSRVYLHTLAESICKLIFPEFERLNVALQRTLAKHKIKESRKSLEREDFEKTIAEQAVAAGVPVEVIKESLGEEVFKICYEEDENILGVVGGTLKDFLNSFSTLLKQSSHCQEAGKRGRLEDASILCLDKEDDFLHVYYFFPKRTTSLILPGIIKAAAHVLYETEVEVSLMPPCFHNDCSEFVNQPYLLYSVHMKSTKPSLSPSKPQSSLVIPTSLFCKTFPFHFMFDKDMTILQFGNGIRRLMNRRDFQGKPNFEEYFEILTPKINQTFSGIMTMLNMQFVVRVRRWDNSVKKSSRVMDLKGQMIYIVESSAILFLGSPCVDRLEDFTGRGLYLSDIPIHNALRDVVLIGEQARAQDGLKKRLGKLKATLEQAHQALEEEKKKTVDLLCSIFPCEVAQQLWQGQVVQAKKFSNVTMLFSDIVGFTAICSQCSPLQVITMLNALYTRFDQQCGELDVYKVETIGDAYCVAGGLHKESDTHAVQIALMALKMMELSDEVMSPHGEPIKMRIGLHSGSVFAGVVGVKMPRYCLFGNNVTLANKFESCSVPRKINVSPTTYRLLKDCPGFVFTPRSREELPPNFPSEIPGICHFLDAYQQGTNSKPCFQKKDVEDGNANFLGKASGID **593 - 606:      RYCLFgnnVtlanK**  **>sp\|Q02108-2\|GCYA1_HUMAN**(624 aa) Guanylate cyclase soluble subunit alpha-1 (GCS-alpha-1) (EC 4.6.1.2) (Guanylate cyclase soluble subunit alpha-3) (GCS-alpha-3) (Soluble guanylate cyclase large subunit) MFCTKLKDLKITGECPFSLLAPGQVPNESSEEAAGSSESCKATVPICQDIPEKNIQESLPQRKTSRSRVYLHTLAESICKLIFPEFERLNVALQRTLAKHKIKESRKSLEREDFEKTIAEQAVAAGVPVEVIKESLGEEVFKICYEEDENILGVVGGTLKDFLNSFSTLLKQSSHCQEAGKRGRLEDASILCLDKEDDFLHVYYFFPKRTTSLILPGIIKAAAHVLYETEVEVSLMPPCFHNDCSEFVNQPYLLYSVHMKSTKPSLSPSKPQSSLVIPTSLFCKTFPFHFMFDKDMTILQFGNGIRRLMNRRDFQGKPNFEEYFEILTPKINQTFSGIMTMLNMQFVVRVRRWDNSVKKSSRVMDLKGQMIYIVESSAILFLGSPCVDRLEDFTGRGLYLSDIPIHNALRDVVLIGEQARAQDGLKKRLGKLKATLEQAHQALEEEKKKTVDLLCSIFPCEVAQQLWQGQVVQAKKFSNVTMLFSDIVGFTAICSQCSPLQVITMLNALYTRFDQQCGELDVYKVETIGDAYCVAGGLHKESDTHAVQIALMALKMMELSDEVMSPHGEPIKMRIGLHSGSVFAGVVGVKMPRYCLFGNNVTLANKFESCSVPRKINVSPTTYR **593 - 606:      RYCLFgnnVtlanK** |
| **>sp\|P33402\|GCYA2_HUMAN** (732 aa) Guanylate cyclase soluble subunit alpha-2 (GCS-alpha-2) (EC 4.6.1.2) MSRRKISSESFSSLGSDYLETSPEEEGECPLSRLCWNGSRSPPGPLEPSPAAAAAAAAPAPTPAASAAAAAATAGARRVQRRRRVNLDSLGESISRLTAPSPQTIQQTLKRTLQYYEHQVIGYRDAEKNFHNISNRCSYADHSNKEEIEDVSGILQCTANILGLKFEEIQKRFGEEFFNICFHENERVLRAVGGTLQDFFNGFDALLEHIRTSFGKQATLESPSFLCKELPEGTLMLHYFHPHHIVGFAMLGMIKAAGKKIYRLDVEVEQVANEKLCSDVSNPGNCSCLTFLIKECENTNIMKNLPQGTSQVPADLRISINTFCRAFPFHLMFDPSMSVLQLGEGLRKQLRCDTHKVLKFEDCFEIVSPKVNATFERVLLRLSTPFVIRTKPEASGSENKDKVMEVKGQMIHVPESNSILFLGSPCVDKLDELMGRGLHLSDIPIHDATRDVILVGEQAKAQDGLKKRMDKLKATLERTHQALEEEKKKTVDLLYSIFPGDVAQQLWQGQQVQARKFDDVTMLFSDIVGFTAICAQCTPMQVISMLNELYTRFDHQCGFLDIYKVETIGDAYCVAAGLHRKSLCHAKPIALMALKMMELSEEVLTPDGRPIQMRIGIHSGSVLAGVVGVRMPRYCLFGNNVTLASKFESGSHPRRINVSPTTYQLLKREESFTFIPRSREELPDNFPKEIPGICYFLEVRTGPKPPKPSLSSSRIKKVSYNIGTMFLRETSL **633 - 646:      RYCLFgnnVtlasK**  **>sp\|P33402-2\|GCYA2_HUMAN** (763 aa) Guanylate cyclase soluble subunit alpha-2 (GCS-alpha-2) (EC 4.6.1.2). MSRRKISSESFSSLGSDYLETSPEEEGECPLSRLCWNGSRSPPGPLEPSPAAAAAAAAPAPTPAASAAAAAATAGARRVQRRRRVNLDSLGESISRLTAPSPQTIQQTLKRTLQYYEHQVIGYRDAEKNFHNISNRCSYADHSNKEEIEDVSGILQCTANILGLKFEEIQKRFGEEFFNICFHENERVLRAVGGTLQDFFNGFDALLEHIRTSFGKQATLESPSFLCKELPEGTLMLHYFHPHHIVGFAMLGMIKAAGKKIYRLDVEVEQVANEKLCSDVSNPGNCSCLTFLIKECENTNIMKNLPQGTSQVPADLRISINTFCRAFPFHLMFDPSMSVLQLGEGLRKQLRCDTHKVLKFEDCFEIVSPKVNATFERVLLRLSTPFVIRTKPEASGSENKDKVMEVKGQMIHVPESNSILFLGSPCVDKLDELMGRGLHLSDIPIHDATRDVILVGEQAKAQDGLKKRMDKLKATLERTHQALEEEKKKTVDLLYSIFPGDVAQQLWQGQQVQARKFDDVTMLFSDIVGFTAICAQCTPMQVISMLNELYTRFDHQCGFLDIYKVETIGDAYCVAAGLHRKSLCHAKPIALMALKMMELSEEVLTPDGRPIQPQRSELLFSFPVSIQLVPDQHQSETDLGTEKMRIGIHSGSVLAGVVGVRMPRYCLFGNNVTLASKFESGSHPRRINVSPTTYQLLKREESFTFIPRSREELPDNFPKEIPGICYFLEVRTGPKPPKPSLSSSRIKKVSYNIGTMFLRETSL **664 - 677:      RYCLFgnnVtlasK**  **>sp\|P33402-3\|GCYA2_HUMAN** (753 aa) Guanylate cyclase soluble subunit alpha-2 (GCS-alpha-2) (EC 4.6.1.2). MSRRKISSESFSSLGSDYLETSPEEEGECPLSRLCWNGSRSPPGPLEPSPAAAAAAAAPAPTPAASAAAAAATAGARRVQRRRRVNLDSLGESISRLTAPSPQTIQQTLKRTLQYYEHQVIGYRDAEKNFHNISNRCSYADHSNKEEIEDVSGILQCTANILGLKFEEIQKRFGEEFFNICFHENERVLRAVGGTLQDFFNGFDALLEHIRTSFGKQATLESPSFLCKELPEGTLMLHYFHPHHIVGFAMLGMIKAAGKKIYRLDVEVEQVANEKLCSDVSNPGNCSCLTFLIKECENTNIMKNLPQGTSQVPADLRISINTFCRAFPFHLMFDPSMSVLQLGEGLRKQLRCDTHKVLKFEDCFEIVSPKVNATFERVLLRLSTPFVIRTKPEASGSENKDKSKHVTEGHLTQLSVAGFNSLEVMEVKGQMIHVPESNSILFLGSPCVDKLDELMGRGLHLSDIPIHDATRDVILVGEQAKAQDGLKKRMDKLKATLERTHQALEEEKKKTVDLLYSIFPGDVAQQLWQGQQVQARKFDDVTMLFSDIVGFTAICAQCTPMQVISMLNELYTRFDHQCGFLDIYKVETIGDAYCVAAGLHRKSLCHAKPIALMALKMMELSEEVLTPDGRPIQMRIGIHSGSVLAGVVGVRMPRYCLFGNNVTLASKFESGSHPRRINVSPTTYQLLKREESFTFIPRSREELPDNFPKEIPGICYFLEVRTGPKPPKPSLSSSRIKKVSYNIGTMFLRETSL **654 - 667:      RYCLFgnnVtlasK** |
| **>sp\|Q02153\|GCYB1_HUMAN** (619 aa) Guanylate cyclase soluble subunit beta-1 (GCS-beta-1) (EC 4.6.1.2) (Guanylate cyclase soluble subunit beta-3) (GCS-beta-3) (Soluble guanylate cyclase small subunit) MYGFVNHALELLVIRNYGPEVWEDIKKEAQLDEEGQFLVRIIYDDSKTYDLVAAASKVLNLNAGEILQMFGKMFFVFCQESGYDTILRVLGSNVREFLQNLDALHDHLATIYPGMRAPSFRCTDAEKGKGLILHYYSEREGLQDIVIGIIKTVAQQIHGTEIDMKVIQQRNEECDHTQFLIEEKESKEEDFYEDLDRFEENGTQESRISPYTFCKAFPFHIIFDRDLVVTQCGNAIYRVLPQLQPGNCSLLSVFSLVRPHIDISFHGILSHINTVFVLRSKEGLLDVEKLECEDELTGTEISCLRLKGQMIYLPEADSILFLCSPSVMNLDDLTRRGLYLSDIPLHDATRDLVLLGEQFREEYKLTQELEILTDRLQLTLRALEDEKKKTDTLLYSVLPPSVANELRHKRPVPAKRYDNVTILFSGIVGFNAFCSKHASGEGAMKIVNLLNDLYTRFDTLTDSRKNPFVYKVETVGDKYMTVSGLPEPCIHHARSICHLALDMMEIAGQVQVDGESVQITIGIHTGEVVTGVIGQRMPRYCLFGNTVNLTSRTETTGEKGKINVSEYTYRCLMSPENSDPQFHLEHRGPVSMKGKKEPMQVWFLSRKNTGTEETKQDDD **539 - 552:      RYCLFgntVnltsR**  **>sp\|Q02153-2\|GCYB1_HUMAN** (586 aa) Guanylate cyclase soluble subunit beta-1 (GCS-beta-1) (EC 4.6.1.2) (Guanylate cyclase soluble subunit beta-3) (GCS-beta-3) (Soluble guanylate cyclase small subunit) MYGFVNHALELLVIRNYGPEVWEDIKKEAQLDEEGQFLVRIIYDDSKTYDLVAAASKVLNLNAGEILQMFGKMFFVFCQESGYDTILRVLGSNVREFLQNLDALHDHLATIYPGMRAPSFRCTDAEKGKGLILHYYSEREGLQDIVIGIIKTVAQQIHGTEIDMKVIQQRNEECDHTQFLIEEKESKEEDFYEDLDRFEENGTQESRISPYTFCKAFPFHIIFDRDLVVTQCGNAIYRVLPQLQPGNCSLLSVFSLVRPHIDISFHGILSHINTVFVLRSKEGLLDVEKLECEDELTGTEISCLRLKGQMIYLPEADSILFLCSPSVMNLDDLTRRGLYLSDIPLHDATRDLVLLGEQFREEYKLTQELEILTDRLQLTLRALEDEKKKTDTGIVGFNAFCSKHASGEGAMKIVNLLNDLYTRFDTLTDSRKNPFVYKVETVGDKYMTVSGLPEPCIHHARSICHLALDMMEIAGQVQVDGESVQITIGIHTGEVVTGVIGQRMPRYCLFGNTVNLTSRTETTGEKGKINVSEYTYRCLMSPENSDPQFHLEHRGPVSMKGKKEPMQVWFLSRKNTGTEETKQDDD **506 - 519:      RYCLFgntVnltsR**  **>sp\|Q02153-3\|GCYB1_HUMAN** (599 aa) Guanylate cyclase soluble subunit beta-1 (GCS-beta-1) (EC 4.6.1.2) (Guanylate cyclase soluble subunit beta-3) (GCS-beta-3) (Soluble guanylate cyclase small subunit) MLMCFIKEAQLDEEGQFLVRIIYDDSKTYDLVAAASKVLNLNAGEILQMFGKMFFVFCQESGYDTILRVLGSNVREFLQNLDALHDHLATIYPGMRAPSFRCTDAEKGKGLILHYYSEREGLQDIVIGIIKTVAQQIHGTEIDMKVIQQRNEECDHTQFLIEEKESKEEDFYEDLDRFEENGTQESRISPYTFCKAFPFHIIFDRDLVVTQCGNAIYRVLPQLQPGNCSLLSVFSLVRPHIDISFHGILSHINTVFVLRSKEGLLDVEKLECEDELTGTEISCLRLKGQMIYLPEADSILFLCSPSVMNLDDLTRRGLYLSDIPLHDATRDLVLLGEQFREEYKLTQELEILTDRLQLTLRALEDEKKKTDTLLYSVLPPSVANELRHKRPVPAKRYDNVTILFSGIVGFNAFCSKHASGEGAMKIVNLLNDLYTRFDTLTDSRKNPFVYKVETVGDKYMTVSGLPEPCIHHARSICHLALDMMEIAGQVQVDGESVQITIGIHTGEVVTGVIGQRMPRYCLFGNTVNLTSRTETTGEKGKINVSEYTYRCLMSPENSDPQFHLEHRGPVSMKGKKEPMQVWFLSRKNTGTEETKQDDD **519 - 532:      RYCLFgntVnltsR** |
| **>sp\|O75343\|GCYB2_HUMAN** (617 aa) Guanylate cyclase soluble subunit beta-2 (GCS-beta-2) (EC 4.6.1.2) MSGYDRMLRTLGGNLMEFIENLDALHSYLALSYQEMNAPSFRVERGADGKMFLHYYSDRSGLCHIVPGIIEAVAKDFFDIDVIMDILDMNEEVERTGKKEHVVFLIVQKAHRKMRKTKPKRLQDSQGMERDQEALQAAFLKMKEKYLNVSACPVKKSHWDVVRSIVMFGKGHLMNTFEPIYPERLWIEEKTFCNAFPFHIVFDESLQVKQARVNIQKYVPGLQTQNIQLDEYFSIIHPQVTFNIFSIRRFINSQFVLKTRREMMPVAWQSRTTLKLQGQMIWMESMWCMVYLCSPKLRSLQELEELNMHLSDIAPNDTTRDLILLNQQRLAEIELSNQLERKKEELQVLSKHLAIEKKKTETLLYAMLPKHVANQLREGKKVAAGEFKSCTILFSDVVTFTNICTACEPIQIVNVLNSMYSKFDRLTSVHAVYKVETIGDAYMVVGGVPVPIGNHAQRVANFALGMRISAKEVTNPVTGEPIQLRVGIHTGPVLADVVGDKMPRYCLFGDTVNTASRMESHGLPNKVHLSPTAYRALKNQGFKIIERGEIEVKGKGRMTTYFLIQNLNATEDEIMGRSKTPVDHKGSTQKASLPTTKLQGSVQPSCPEHSSLASWLL **504 - 517:      RYCLFgdtVntasR** |
| **>sp\|P25092\|GUC2C_HUMAN** (1073 aa) Guanylyl cyclase C (GC-C) (EC 4.6.1.2) (Heat-stable enterotoxin receptor) (STA receptor) (hSTAR) (Intestinal guanylate cyclase) MKTLLLDLALWSLLFQPGWLSFSSQVSQNCHNGSYEISVLMMGNSAFAEPLKNLEDAVNEGLEIVRGRLQNAGLNVTVNATFMYSDGLIHNSGDCRSSTCEGLDLLRKISNAQRMGCVLIGPSCTYSTFQMYLDTELSYPMISAGSFGLSCDYKETLTRLMSPARKLMYFLVNFWKTNDLPFKTYSWSTSYVYKNGTETEDCFWYLNALEASVSYFSHELGFKVVLRQDKEFQDILMDHNRKSNVIIMCGGPEFLYKLKGDRAVAEDIVIILVDLFNDQYFEDNVTAPDYMKNVLVLTLSPGNSLLNSSFSRNLSPTKRDFALAYLNGILLFGHMLKIFLENGENITTPKFAHAFRNLTFEGYDGPVTLDDWGDVDSTMVLLYTSVDTKKYKVLLTYDTHVNKTYPVDMSPTFTWKNSKLPNDITGRGPQILMIAVFTLTGAVVLLLLVALLMLRKYRKDYELRQKKWSHIPPENIFPLETNETNHVSLKIDDDKRRDTIQRLRQCKYDKKRVILKDLKHNDGNFTEKQKIELNKLLQIDYYNLTKFYGTVKLDTMIFGVIEYCERGSLREVLNDTISYPDGTFMDWEFKISVLYDIAKGMSYLHSSKTEVHGRLKSTNCVVDSRMVVKITDFGCNSILPPKKDLWTAPEHLRQANISQKGDVYSYGIIAQEIILRKETFYTLSCRDRNEKIFRVENSNGMKPFRPDLFLETAEEKELEVYLLVKNCWEEDPEKRPDFKKIETTLAKIFGLFHDQKNESYMDTLIRRLQLYSRNLEHLVEERTQLYKAERDRADRLNFMLLPRLVVKSLKEKGFVEPELYEEVTIYFSDIVGFTTICKYSTPMEVVDMLNDIYKSFDHIVDHHDVYKVETIGDAYMVASGLPKRNGNRHAIDIAKMALEILSFMGTFELEHLPGLPIWIRIGVHSGPCAAGVVGIKMPRYCLFGDTVNTASRMESTGLPLRIHVSGSTIAILKRTECQFLYEVRGETYLKGRGNETTYWLTGMKDQKFNLPTPPTVENQQRLQAEFSDMIANSLQKRQAAGIRSQKPRRVASYKKGTLEYLQLNTTDKESTYF **939 - 952:      RYCLFgdtVntasR** |
| **>sp\|Q02846\|GUC2D_HUMAN** (1103 aa) Retinal guanylyl cyclase 1 (RETGC-1) (EC 4.6.1.2) (CG-E) (Guanylate cyclase 2D, retinal) (Rod outer segment membrane guanylate cyclase) (ROS-GC)  MTACARRAGGLPDPGLCGPAWWAPSLPRLPRALPRLPLLLLLLLLQPPALSAVFTVGVLGPWACDPIFSRARPDLAARLAAARLNRDPGLAGGPRFEVALLPEPCRTPGSLGAVSSALARVSGLVGPVNPAACRPAELLAEEAGIALVPWGCPWTQAEGTTAPAVTPAADALYALLRAFGWARVALVTAPQDLWVEAGRSLSTALRARGLPVASVTSMEPLDLSGAREALRKVRDGPRVTAVIMVMHSVLLGGEEQRYLLEAAEELGLTDGSLVFLPFDTIHYALSPGPEALAALANSSQLRRAHDAVLTLTRHCPSEGSVLDSLRRAQERRELPSDLNLQQVSPLFGTIYDAVFLLARGVAEARAAAGGRWVSGAAVARHIRDAQVPGFCGDLGGDEEPPFVLLDTDAAGDRLFATYMLDPARGSFLSAGTRMHFPRGGSAPGPDPSCWFDPNNICGGGLEPGLVFLGFLLVVGMGLAGAFLAHYVRHRLLHMQMVSGPNKIILTVDDITFLHPHGGTSRKVAQGSRSSLGARSMSDIRSGPSQHLDSPNIGVYEGDRVWLKKFPGDQHIAIRPATKTAFSKLQELRHENVALYLGLFLARGAEGPAALWEGNLAVVSEHCTRGSLQDLLAQREIKLDWMFKSSLLLDLIKGIRYLHHRGVAHGRLKSRNCIVDGRFVLKITDHGHGRLLEAQKVLPEPPRAEDQLWTAPELLRDPALERRGTLAGDVFSLAIIMQEVVCRSAPYAMLELTPEEVVQRVRSPPPLCRPLVSMDQAPVECILLMKQCWAEQPELRPSMDHTFDLFKNINKGRKTNIIDSMLRMLEQYSSNLEDLIRERTEELELEKQKTDRLLTQMLPPSVAEALKTGTPVEPEYFEQVTLYFSDIVGFTTISAMSEPIEVVDLLNDLYTLFDAIIGSHDVYKVETIGDAYMVASGLPQRNGQRHAAEIANMSLDILSAVGTFRMRHMPEVPVRIRIGLHSGPCVAGVVGLTMPRYCLFGDTVNTASRMESTGLPYRIHVNLSTVGILRALDSGYQVELRGRTELKGKGAEDTFWLVGRRGFNKPIPKPPDLQPGSSNHGISLQEIPPERRRKLEKARPGQFS **995 - 1008:     RYCLFgdtVntasR** |
| **>sp\|P51841\|GUC2F_HUMAN** (1108 aa) Retinal guanylyl cyclase 2 (RETGC-2) (EC 4.6.1.2) (Guanylate cyclase 2F, retinal) (Guanylate cyclase F) (GC-F) (Rod outer segment membrane guanylate cyclase 2) (ROS-GC2) MFLGLGRFSRLVLWFAAFRKLLGHHGLASAKFLWCLCLLSVMSLPQQVWTLPYKIGVVGPWACDSLFSKALPEVAARLAIERINRDPSFDLSYSFEYVILNEDCQTSRALSSFISHHQMASGFIGPTNPGYCEAASLLGNSWDKGIFSWACVNYELDNKISYPTFSRTLPSPIRVLVTVMKYFQWAHAGVISSDEDIWVHTANRVASALRSHGLPVGVVLTTGQDSQSMRKALQRIHQADRIRIIIMCMHSALIGGETQMHLLECAHDLKMTDGTYVFVPYDALLYSLPYKHTPYRVLRNNPKLREAYDAVLTITVESQEKTFYQAFTEAAARGEIPEKLEFDQVSPLFGTIYNSIYFIAQAMNNAMKENGQAGAASLVQHSRNMQFHGFNQLMRTDSNGNGISEYVILDTNLKEWELHSTYTVDMEMELLRFGGTPIHFPGGRPPRADAKCWFAEGKICHGGIDPAFAMMVCLTLLIALLSINGFAYFIRRRINKIQLIKGPNRILLTLEDVTFINPHFGSKRGSRASVSFQITSEVQSGRSPRLSFSSGSLTPATYENSNIAIYEGDWVWLKKFSLGDFGDLKSIKSRASDVFEMMKDLRHENINPLLGFFYDSGMFAIVTEFCSRGSLEDILTNQDVKLDWMFKSSLLLDLIKGMKYLHHREFVHGRLKSRNCVVDGRFVLKVTDYGFNDILEMLRLSEEESSMEELLWTAPELLRAPRGSRLGSFAGDVYSFAIIMQEVMVRGTPFCMMDLPAQEIINRLKKPPPVYRPVVPPEHAPPECLQLMKQCWAEAAEQRPTFDEIFNQFKTFNKGKKTNIIDSMLRMLEQYSSNLEDLIRERTEELEIEKQKTEKLLTQMLPPSVAESLKKGCTVEPEGFDLVTLYFSDIVGFTTISAMSEPIEVVDLLNDLYTLFDAIIGSHDVYKVETIGDAYMVASGLPKRNGSRHAAEIANMSLDILSSVGTFKMRHMPEVPVRIRIGLHSGPVVAGVVGLTMPRYCLFGDTVNTASRMESTGLPYRIHVSLSTVTILQNLSEGYEVELRGRTELKGKGTEETFWLIGKKGFMKPLPVPPPVDKDGQVGHGLQPVEIAAFQRRKAERQLVRNKP **999 - 1012:     RYCLFgdtVntasR** |
| **>sp\|Q9Y616\|IRAK3_HUMAN** (596 aa) Interleukin-1 receptor-associated kinase 3 (IRAK-3) (IL-1 receptor-associated kinase M) (IRAK-M) (Inactive IL-1 receptor-associated kinase 3) MAGNCGARGALSAHTLLFDLPPALLGELCAVLDSCDGALGWRGLAERLSSSWLDVRHIEKYVDQGKSGTRELLWSWAQKNKTIGDLLQVLQEMGHRRAIHLITNYGAVLSPSEKSYQEGGFPNILFKETANVTVDNVLIPEHNEKGILLKSSISFQNIIEGTRNFHKDFLIGEGEIFEVYRVEIQNLTYAVKLFKQEKKMQCKKHWKRFLSELEVLLLFHHPNILELAAYFTETEKFCLIYPYMRNGTLFDRLQCVGDTAPLPWHIRIGILIGISKAIHYLHNVQPCSVICGSISSANILLDDQFQPKLTDFAMAHFRSHLEHQSCTINMTSSSSKHLWYMPEEYIRQGKLSIKTDVYSFGIVIMEVLTGCRVVLDDPKHIQLRDLLRELMEKRGLDSCLSFLDKKVPPCPRNFSAKLFCLAGRCAATRAKLRPSMDEVLNTLESTQASLYFAEDPPTSLKSFRCPSPLFLENVPSIPVEDDESQNNNLLPSDEGLRIDRMTQKTPFECSQSEVMFLSLDKKPESKRNEEACNMPSSSCEESWFPKYIVPSQDLRPYKVNIDPSSEAPGHSCRSRPVESSCSSKFSWDEYEQYKKE **359 - 372:      SFGIVimeVltgcR**  **>sp\|Q9Y616-2\|IRAK3_HUMAN** (535 aa) Interleukin-1 receptor-associated kinase 3 (IRAK-3) (IL-1 receptor-associated kinase M) (IRAK-M) (Inactive IL-1 receptor-associated kinase 3) MAGNCGARGALSAHTLLFDLPPALLGELCAVLDSCDGALGWRGLGAVLSPSEKSYQEGGFPNILFKETANVTVDNVLIPEHNEKGILLKSSISFQNIIEGTRNFHKDFLIGEGEIFEVYRVEIQNLTYAVKLFKQEKKMQCKKHWKRFLSELEVLLLFHHPNILELAAYFTETEKFCLIYPYMRNGTLFDRLQCVGDTAPLPWHIRIGILIGISKAIHYLHNVQPCSVICGSISSANILLDDQFQPKLTDFAMAHFRSHLEHQSCTINMTSSSSKHLWYMPEEYIRQGKLSIKTDVYSFGIVIMEVLTGCRVVLDDPKHIQLRDLLRELMEKRGLDSCLSFLDKKVPPCPRNFSAKLFCLAGRCAATRAKLRPSMDEVLNTLESTQASLYFAEDPPTSLKSFRCPSPLFLENVPSIPVEDDESQNNNLLPSDEGLRIDRMTQKTPFECSQSEVMFLSLDKKPESKRNEEACNMPSSSCEESWFPKYIVPSQDLRPYKVNIDPSSEAPGHSCRSRPVESSCSSKFSWDEYEQYKKE **298 - 311:      SFGIVimeVltgcR** |
| **>sp\|Q13387\|JIP2_HUMAN** (824 aa) C-Jun-amino-terminal kinase-interacting protein 2 (JIP-2) (JNK-interacting protein 2) (Islet-brain-2) (IB-2) (JNK MAP kinase scaffold protein 2) (Mitogen-activated protein kinase 8-interacting protein 2) MADRAEMFSLSTFHSLSPPGCRPPQDISLEEFDDEDLSEITDDCGLGLSYDSDHCEKDSLSLGRSEQPHPICSFQDDFQEFEMIDDNEEEDDEDEEEEEEEEEGDGEGQEGGDPGSEAPAPGPLIPSPSVEEPHKHRPTTLRLTTLGAQDSLNNNGGFDLVRPASWQETALCSPAPEALRELPGPLPATDTGPGGAQSPVRPGCDCEGNRPAEPPAPGGTSPSSDPGIEADLRSRSSGGRGGRRSSQELSSPGSDSEDAGGARLGRMISSISETELELSSDGGSSSSGRSSHLTNSIEEASSPASEPEPPREPPRRPAFLPVGPDDTNSEYESGSESEPDLSEDADSPWLLSNLVSRMISEGSSPIRCPGQCLSPAPRPPGEPVSPAGGAAQDSQDPEAAAGPGGVELVDMETLCAPPPPAPAAPRPGPAQPGPCLFLSNPTRDTITPLWAAPGRAARPGRACSAACSEEEDEEDDEEEEDAEDSAGSPGGRGTGPSAPRDASLVYDAVKYTLVVDEHTQLELVSLRRCAGLGHDSEEDSGGEASEEEAGAALLGGGQVSGDTSPDSPDLTFSKKFLNVFVNSTSRSSSTESFGLFSCLVNGEEREQTHRAVFRFIPRHPDELELDVDDPVLVEAEEDDFWFRGFNMRTGERGVFPAFYAHAVPGPAKDLLGSKRSPCWVERFDVQFLGSVEVPCHQGNGILCAAMQKIATARKLTVHLRPPASCDLEISLRGVKLSLSGGGPEFQRCSHFFQMKNISFCGCHPRNSCYFGFITKHPLLSRFACHVFVSQESMRPVAQSVGRAFLEYYQEHLAYACPTEDIYLE **592 - 605:      SFGLFsclVngeeR**  **>sp\|Q13387-2\|JIP2_HUMAN** (797 aa) C-Jun-amino-terminal kinase-interacting protein 2 (JIP-2) (JNK-interacting protein 2) (Islet-brain-2) (IB-2) (JNK MAP kinase scaffold protein 2) (Mitogen-activated protein kinase 8-interacting protein 2) MLPDFPSPSTWAPGLLLPSGPALLSPSVLQDSLSLGRSEQPHPICSFQDDFQEFEMIDDNEEEDDEDEEEEEEEEEGDGEGQEGGDPGSEAPAPGPLIPSPSVEEPHKHRPTTLRLTTLGAQDSLNNNGGFDLVRPASWQETALCSPAPEALRELPGPLPATDTGPGGAQSPVRPGCDCEGNRPAEPPAPGGTSPSSDPGIEADLRSRSSGGRGGRRSSQELSSPGSDSEDAGGARLGRMISSISETELELSSDGGSSSSGRSSHLTNSIEEASSPASEPEPPREPPRRPAFLPVGPDDTNSEYESGSESEPDLSEDADSPWLLSNLVSRMISEGSSPIRCPGQCLSPAPRPPGEPVSPAGGAAQDSQDPEAAAGPGGVELVDMETLCAPPPPAPAAPRPGPAQPGPCLFLSNPTRDTITPLWAAPGRAARPGRACSAACSEEEDEEDDEEEEDAEDSAGSPGGRGTGPSAPRDASLVYDAVKYTLVVDEHTQLELVSLRRCAGLGHDSEEDSGGEASEEEAGAALLGGGQVSGDTSPDSPDLTFSKKFLNVFVNSTSRSSSTESFGLFSCLVNGEEREQTHRAVFRFIPRHPDELELDVDDPVLVEAEEDDFWFRGFNMRTGERGVFPAFYAHAVPGPAKDLLGSKRSPCWVERFDVQFLGSVEVPCHQGNGILCAAMQKIATARKLTVHLRPPASCDLEISLRGVKLSLSGGGPEFQRCSHFFQMKNISFCGCHPRNSCYFGFITKHPLLSRFACHVFVSQESMRPVAQSVGRAFLEYYQEHLAYACPTEDIYLE **565 - 578:      SFGLFsclVngeeR**  **>sp\|Q13387-3\|JIP2_HUMAN** (598 aa) C-Jun-amino-terminal kinase-interacting protein 2 (JIP-2) (JNK-interacting protein 2) (Islet-brain-2) (IB-2) (JNK MAP kinase scaffold protein 2) (Mitogen-activated protein kinase 8-interacting protein 2) MLPDFPSPSTWAPGLLLPSGPALLSPSVLQDSLSLGRSEQPHPICSFQDDFQEFEMIDDNEEEDDEDEEEEEEEEEGDGEGQEGGDPGSEAPAPGPLIPSPSVEEPHKHRPTTLRLTTLGAQDSQDPEAAAGPGGVELVDMETLNPTRDTITPLWAAPGRAARPGRACSAACSEEEDEEDDEEEEDAEDSAGSPGGRGTGPSAPRDASLVYDAVKYTLVVDEHTQLELVSLRRCAGLGHDSEEDSGGEASEEEAGAALLGGGQVSGDTSPDSPDLTFSKKFLNVFVNSTSRSSSTESFGLFSCLVNGEEREQTHRAVFRFIPRHPDELELDVDDPVLVEAEEDDFWFRGFNMRTGERGVFPAFYAHAVPGPAKDLLGSKRSPCWVERFDVQFLGSVEVPCHQGNGILCAAMQKIATARKLTVHLRPPASCDLEISLRGVKLSLSGGGPEFQRCSHFFQMKNISFCGCHPRNSCEAPQGAAFQWERGVDRKRVLQTRGNVQPHLGAGQGAALNRATEGSSTGSEKGEWTPLVIMELTQSVNSCYFGFITKHPLLSRFACHVFVSQESMRPVAQSVGRAFLEYYQEHLAYACPTEDIYLE **297 - 310:      SFGLFsclVngeeR**  **>sp\|Q13387-4\|JIP2_HUMAN**  (443 aa) C-Jun-amino-terminal kinase-interacting protein 2 (JIP-2) (JNK-interacting protein 2) (Islet-brain-2) (IB-2) (JNK MAP kinase scaffold protein 2) (Mitogen-activated protein kinase 8-interacting protein 2) MADRAEMFSLSTFHSLSPPGCRPPQDISLEEFDDEDLSEITDDCGLGLSYDSDHCEKDSLSLGRSEQPHPICSFQDDFQEFEMIDDNEEEDEEDDEEEEDAEDSAGSPGGRGTGPSAPRDASLVYDAVKYTLVVDEHTQLELVSLRRCAGLGHDSEEDSGGEASEEEAGAALLGGGQVSGDTSPDSPDLTFSKKFLNVFVNSTSRSSSTESFGLFSCLVNGEEREQTHRAVFRFIPRHPDELELDVDDPVLVEAEEDDFWFRGFNMRTGERGVFPAFYAHAVPGPAKDLLGSKRSPCWVERFDVQFLGSVEVPCHQGNGILCAAMQKIATARKLTVHLRPPASCDLEISLRGVKLSLSGGGPEFQRCSHFFQMKNISFCGCHPRNSCYFGFITKHPLLSRFACHVFVSQESMRPVAQSVGRAFLEYYQEHLAYACPTEDIYLE **211 - 224:      SFGLFsclVngeeR** |
| **>sp\|Q6ZRQ5\|MMS22_HUMAN** (1243 aa) Protein MMS22-like (Methyl methanesulfonate-sensitivity protein 22-like) MENCSAASTFLTDSLELELGTEWCKPPYFSCAVDNRGGGKHFSGESYLCSGALKRLILNLDPLPTNFEEDTLEIFGIQWVTETALVNSSRELFHLFRQQLYNLETLLQSSCDFGKVSTLHCKADNIRQQCVLFLHYVKVFIFRYLKVQNAESHVPVHPYEALEAQLPSVLIDELHGLLLYIGHLSELPSVNIGAFVNQNQIKLFPPSWHLLHLHLDIHWLVLEILYMLGEKLKQVVYGHQFMNLASDNLTNISLFEEHCETLLCDLISLSLNRYDKVRSSESLMSDQCPCLCIKELWVLLIHLLDHRSKWFVSESFWNWLNKLLKTLLEKSSDRRRSSMPVIQSRDPLGFSWWIITHVASFYKFDRHGVPDEMRKVESNWNFVEELLKKSISVQGVILEEQLRMYLHCCLTLCDFWEPNIAIVTILWEYYSKNLNSSFSISWLPFKGLANTMKSPLSMLEMVKTCCCDKQDQELYKSSSSYTIFLCILAKVVKKAMKSNGPHPWKQVKGRIYSKFHQKRMEELTEVGLQNFFSLFLLLAAVAEVEDVASHVLDLLNFLKPAFVTSQRALIWKGHMAFLLMYAQKNLDIGVLAEKFSCAFREKAKEFLVSKNEEMVQRQTIWTLLSIYIDGVQEVFETSYCLYPSHEKLLNDGFSMLLRACRESELRTVLSFLQAVLARIRSMHQQLCQELQRDNVDLFVQSSLSAKERHLAAVASALWRHFFSFLKSQRMSQVVPFSQLADAAADFTLLAMDMPSTAPSDFQPQPVISIIQLFGWDDIICPQVVARYLSHVLQNSTLCEALSHSGYVSFQALTVRSWIRCVLQMYIKNLSGPDDLLIDKNLEEAVEKEYMKQLVKLTRLLFNLSEVKSIFSKAQVEYLSISEDPKKALVRFFEAVGVTYGNVQTLSDKSAMVTKSLEYLGEVLKYIKPYLGKKVFSAGLQLTYGMMGILVKSWAQIFATSKAQKLLFRIIDCLLLPHAVLQQEKELPAPMLSAIQKSLPLYLQGMCIVCCQSQNPNAYLNQLLGNVIEQYIGRFLPASPYVSDLGQHPVLLALRNTATIPPISSLKKCIVQVIRKSYLEYKGSSPPPRLASILAFILQLFKETNTDIYEVELLLPGILKCLVLVSEPQVKRLATENLQYMVKACQVGSEEEPSSQLTSVFRQFIQDYGMRYYYQVYSILETVATLDQQVVIHLISTLTQSLKDSEQKWGLGRNIAQREAYSKLLSHLGQMGQDEMQRLENDNT **480 - 493:      SYTIFlciLakvvK** |
| **>sp\|O95563\|MPC2_HUMAN** (127 aa) Mitochondrial pyruvate carrier 2 (Brain protein 44) MSAAGARGLRATYHRLLDKVELMLPEKLRPLYNHPAGPRTVFFWAPIMKWGLVCAGLADMARPAEKLSTAQSAVLMATGFIWSRYSLVIIPKNWSLFAVNFFVGAAGASQLFRIWRYNQELKAKAHK **49 - 62:        KWGLVcagLadmaR** |
| **>sp\|A0A3B3IT52\|MSD5_HUMAN** (213 aa) Putative uncharacterized protein MSANTD5 (Myb/SANT DNA binding domain-containing protein 5) MEIVILPTETTINIQKMEQENTAQGSEKPSVQSVKPWSDQEIRSFLQEWEFLEREVYRVKKKYHIVSKAIAQRLKQRGINKSWKECLQMLISLQDLYFTIQEANQRPRCQPLPCPYGEALHRILGYRWKISVFSGPPCADVVNLAPPEHPPQAYGVPIVFQEPMWAPTPVIYVENPQVPGWEPWNMNGHVPYMYPALPPAAPGPLTQWAISTD **62 - 75:        KYHIVskaIaqrlK** |
| **>sp\|P04629\|NTRK1_HUMAN** (796 aa) High affinity nerve growth factor receptor (EC 2.7.10.1) (Neurotrophic tyrosine kinase receptor type 1) (TRK1-transforming tyrosine kinase protein) (Tropomyosin-related kinase A) (Tyrosine kinase receptor) (Tyrosine kinase receptor A) (Trk-A) (gp140trk) (p140-TrkA) MLRGGRRGQLGWHSWAAGPGSLLAWLILASAGAAPCPDACCPHGSSGLRCTRDGALDSLHHLPGAENLTELYIENQQHLQHLELRDLRGLGELRNLTIVKSGLRFVAPDAFHFTPRLSRLNLSFNALESLSWKTVQGLSLQELVLSGNPLHCSCALRWLQRWEEEGLGGVPEQKLQCHGQGPLAHMPNASCGVPTLKVQVPNASVDVGDDVLLRCQVEGRGLEQAGWILTELEQSATVMKSGGLPSLGLTLANVTSDLNRKNVTCWAENDVGRAEVSVQVNVSFPASVQLHTAVEMHHWCIPFSVDGQPAPSLRWLFNGSVLNETSFIFTEFLEPAANETVRHGCLRLNQPTHVNNGNYTLLAANPFGQASASIMAAFMDNPFEFNPEDPIPVSFSPVDTNSTSGDPVEKKDETPFGVSVAVGLAVFACLFLSTLLLVLNKCGRRNKFGINRPAVLAPEDGLAMSLHFMTLGGSSLSPTEGKGSGLQGHIIENPQYFSDACVHHIKRRDIVLKWELGEGAFGKVFLAECHNLLPEQDKMLVAVKALKEASESARQDFQREAELLTMLQHQHIVRFFGVCTEGRPLLMVFEYMRHGDLNRFLRSHGPDAKLLAGGEDVAPGPLGLGQLLAVASQVAAGMVYLAGLHFVHRDLATRNCLVGQGLVVKIGDFGMSRDIYSTDYYRVGGRTMLPIRWMPPESILYRKFTTESDVWSFGVVLWEIFTYGKQPWYQLSNTEAIDCITQGRELERPRACPPEVYAIMRGCWQREPQQRHSIKDVHARLQALAQAPPVYLDVLG **712 - 725:      SFGVVlweIftygK**  **>sp\|P04629-2\|NTRK1_HUMAN** (790 aa) High affinity nerve growth factor receptor (EC 2.7.10.1) (Neurotrophic tyrosine kinase receptor type 1) (TRK1-transforming tyrosine kinase protein) (Tropomyosin-related kinase A) (Tyrosine kinase receptor) (Tyrosine kinase receptor A) (Trk-A) (gp140trk) (p140-TrkA) MLRGGRRGQLGWHSWAAGPGSLLAWLILASAGAAPCPDACCPHGSSGLRCTRDGALDSLHHLPGAENLTELYIENQQHLQHLELRDLRGLGELRNLTIVKSGLRFVAPDAFHFTPRLSRLNLSFNALESLSWKTVQGLSLQELVLSGNPLHCSCALRWLQRWEEEGLGGVPEQKLQCHGQGPLAHMPNASCGVPTLKVQVPNASVDVGDDVLLRCQVEGRGLEQAGWILTELEQSATVMKSGGLPSLGLTLANVTSDLNRKNVTCWAENDVGRAEVSVQVNVSFPASVQLHTAVEMHHWCIPFSVDGQPAPSLRWLFNGSVLNETSFIFTEFLEPAANETVRHGCLRLNQPTHVNNGNYTLLAANPFGQASASIMAAFMDNPFEFNPEDPIPDTNSTSGDPVEKKDETPFGVSVAVGLAVFACLFLSTLLLVLNKCGRRNKFGINRPAVLAPEDGLAMSLHFMTLGGSSLSPTEGKGSGLQGHIIENPQYFSDACVHHIKRRDIVLKWELGEGAFGKVFLAECHNLLPEQDKMLVAVKALKEASESARQDFQREAELLTMLQHQHIVRFFGVCTEGRPLLMVFEYMRHGDLNRFLRSHGPDAKLLAGGEDVAPGPLGLGQLLAVASQVAAGMVYLAGLHFVHRDLATRNCLVGQGLVVKIGDFGMSRDIYSTDYYRVGGRTMLPIRWMPPESILYRKFTTESDVWSFGVVLWEIFTYGKQPWYQLSNTEAIDCITQGRELERPRACPPEVYAIMRGCWQREPQQRHSIKDVHARLQALAQAPPVYLDVLG **706 - 719:      SFGVVlweIftygK**  **>sp\|P04629-3\|NTRK1_HUMAN** (760 aa) High affinity nerve growth factor receptor (EC 2.7.10.1) (Neurotrophic tyrosine kinase receptor type 1) (TRK1-transforming tyrosine kinase protein) (Tropomyosin-related kinase A) (Tyrosine kinase receptor) (Tyrosine kinase receptor A) (Trk-A) (gp140trk) (p140-TrkA) MKEAALICLAPSVPPILTVKSWDTMQLRAARSRCTNLLAASYIENQQHLQHLELRDLRGLGELRNLTIVKSGLRFVAPDAFHFTPRLSRLNLSFNALESLSWKTVQGLSLQELVLSGNPLHCSCALRWLQRWEEEGLGGVPEQKLQCHGQGPLAHMPNASCGVPTLKVQVPNASVDVGDDVLLRCQVEGRGLEQAGWILTELEQSATVMKSGGLPSLGLTLANVTSDLNRKNVTCWAENDVGRAEVSVQVNVSFPASVQLHTAVEMHHWCIPFSVDGQPAPSLRWLFNGSVLNETSFIFTEFLEPAANETVRHGCLRLNQPTHVNNGNYTLLAANPFGQASASIMAAFMDNPFEFNPEDPIPDTNSTSGDPVEKKDETPFGVSVAVGLAVFACLFLSTLLLVLNKCGRRNKFGINRPAVLAPEDGLAMSLHFMTLGGSSLSPTEGKGSGLQGHIIENPQYFSDACVHHIKRRDIVLKWELGEGAFGKVFLAECHNLLPEQDKMLVAVKALKEASESARQDFQREAELLTMLQHQHIVRFFGVCTEGRPLLMVFEYMRHGDLNRFLRSHGPDAKLLAGGEDVAPGPLGLGQLLAVASQVAAGMVYLAGLHFVHRDLATRNCLVGQGLVVKIGDFGMSRDIYSTDYYRVGGRTMLPIRWMPPESILYRKFTTESDVWSFGVVLWEIFTYGKQPWYQLSNTEAIDCITQGRELERPRACPPEVYAIMRGCWQREPQQRHSIKDVHARLQALAQAPPVYLDVLG **676 - 689:      SFGVVlweIftygK**  **>sp\|P04629-4\|NTRK1_HUMAN** (698 aa) High affinity nerve growth factor receptor (EC 2.7.10.1) (Neurotrophic tyrosine kinase receptor type 1) (TRK1-transforming tyrosine kinase protein) (Tropomyosin-related kinase A) (Tyrosine kinase receptor) (Tyrosine kinase receptor A) (Trk-A) (gp140trk) (p140-TrkA) MLRGGRRGQLGWHSWAAGPGSLLAWLILASAGAAPCPDACCPHGSSGLRCTRDGALDSLHHLPGAENLTELYIENQQHLQHLELRDLRGLGELRNLTIVKSGLRFVAPDAFHFTPRLSRLNLSFNALESLSWKTVQGLSLQELVLSGNPLHCSCALRWLQRWEEEGLGGVPEQKLQCHGQGPLAHMPNASCVPASVQLHTAVEMHHWCIPFSVDGQPAPSLRWLFNGSVLNETSFIFTEFLEPAANETVRHGCLRLNQPTHVNNGNYTLLAANPFGQASASIMAAFMDNPFEFNPEDPIPDTNSTSGDPVEKKDETPFGVSVAVGLAVFACLFLSTLLLVLNKCGRRNKFGINRPAVLAPEDGLAMSLHFMTLGGSSLSPTEGKGSGLQGHIIENPQYFSDACVHHIKRRDIVLKWELGEGAFGKVFLAECHNLLPEQDKMLVAVKALKEASESARQDFQREAELLTMLQHQHIVRFFGVCTEGRPLLMVFEYMRHGDLNRFLRSHGPDAKLLAGGEDVAPGPLGLGQLLAVASQVAAGMVYLAGLHFVHRDLATRNCLVGQGLVVKIGDFGMSRDIYSTDYYRVGGRTMLPIRWMPPESILYRKFTTESDVWSFGVVLWEIFTYGKQPWYQLSNTEAIDCITQGRELERPRACPPEVYAIMRGCWQREPQQRHSIKDVHARLQALAQAPPVYLDVLG **614 - 627:      SFGVVlweIftygK** |
| **>sp\|Q96KN7\|RPGR1_HUMAN** (1286 aa) X-linked retinitis pigmentosa GTPase regulator-interacting protein 1 (RPGR-interacting protein 1) MSHLVDPTSGDLPVRDIDAIPLVLPASKGKNMKTQPPLSRMNREELEDSFFRLREDHMLVKELSWKQQDEIKRLRTTLLRLTAAGRDLRVAEEAAPLSETARRGQKAGWRQRLSMHQRPQMHRLQGHFHCVGPASPRRAQPRVQVGHRQLHTAGAPVPEKPKRGPRDRLSYTAPPSFKEHATNENRGEVASKPSELVSGSNSIISFSSVISMAKPIGLCMPNSAHIMASNTMQVEEPPKSPEKMWPKDENFEQRSSLECAQKAAELRASIKEKVELIRLKKLLHERNASLVMTKAQLTEVQEAYETLLQKNQGILSAAHEALLKQVNELRAELKEESKKAVSLKSQLEDVSILQMTLKEFQERVEDLEKERKLLNDNYDKLLESMLDSSDSSSQPHWSNELIAEQLQQQVSQLQDQLDAELEDKRKVLLELSREKAQNEDLKLEVTNILQKHKQEVELLQNAATISQPPDRQSEPATHPAVLQENTQIEPSEPKNQEEKKLSQVLNELQVSHAETTLELEKTRDMLILQRKINVCYQEELEAMMTKADNDNRDHKEKLERLTRLLDLKNNRIKQLEGILRSHDLPTSEQLKDVAYGTRPLSLCLETLPAHGDEDKVDISLLHQGENLFELHIHQAFLTSAALAQAGDTQPTTFCTYSFYDFETHCTPLSVGPQPLYDFTSQYVMETDSLFLHYLQEASARLDIHQAMASEHSTLAAGWICFDRVLETVEKVHGLATLIGAGGEEFGVLEYWMRLRFPIKPSLQACNKRKKAQVYLSTDVLGGRKAQEEEFRSESWEPQNELWIEITKCCGLRSRWLGTQPSPYAVYRFFTFSDHDTAIIPASNNPYFRDQARFPVLVTSDLDHYLRREALSIHVFDDEDLEPGSYLGRARVPLLPLAKNESIKGDFNLTDPAEKPNGSIQVQLDWKFPYIPPESFLKPEAQTKGKDTKDSSKISSEEEKASFPSQDQMASPEVPIEAGQYRSKRKPPHGGERKEKEHQVVSYSRRKHGKRIGVQGKNRMEYLSLNILNGNTPEQVNYTEWKFSETNSFIGDGFKNQHEEEEMTLSHSALKQKEPLHPVNDKESSEQGSEVSEAQTTDSDDVIVPPMSQKYPKADSEKMCIEIVSLAFYPEAEVMSDENIKQVYVEYKFYDLPLSETETPVSLRKPRAGEEIHFHFSKVIDLDPQEQQGRRRFLFDMLNGQDPDQGHLKFTVVSDPLDEEKKECEEVGYAYLQLWQILESGRDILEQELDIVSPEDLATPIGRLKVSLQAAAVLHAIYKEMTEDLFS **1208 - 1221:    KFTVVsdpLdeekK**  **>sp\|Q96KN7-2\|RPGR1_HUMAN** (669 aa) X-linked retinitis pigmentosa GTPase regulator-interacting protein 1 (RPGR-interacting protein 1) MTFQHLGENLFELHIHQAFLTSAALAQAGDTQPTTFCTYSFYDFETHCTPLSVGPQPLYDFTSQYVMETDSLFLHYLQEASARLDIHQAMASEHSTLAAGWICFDRVLETVEKVHGLATLIGAGGEEFGVLEYWMRLRFPIKPSLQACNKRKKAQVYLSTDVLGGRKAQEEEFRSESWEPQNELWIEITKCCGLRSRWLGTQPSPYAVYRFFTFSDHDTAIIPASNNPYFRDQARFPVLVTSDLDHYLRREALSIHVFDDEDLEPGSYLGRARVPLLPLAKNESIKGDFNLTDPAEKPNGSIQVQLDWKFPYIPPESFLKPEAQTKGKDTKDSSKISSEEEKASFPSQDQMASPEVPIEAGQYRSKRKPPHGGERKEKEHQVVSYSRRKHGKRIGVQGKNRMEYLSLNILNGNTPEQVNYTEWKFSETNSFIGDGFKNQHEEEEMTLSHSALKQKEPLHPVNDKESSEQGSEVSEAQTTDSDDVIVPPMSQKYPKADSEKMCIEIVSLAFYPEAEVMSDENIKQVYVEYKFYDLPLSETETPVSLRKPRAGEEIHFHFSKVIDLDPQEQQGRRRFLFDMLNGQDPDQGHLKFTVVSDPLDEEKKECEEVGYAYLQLWQILESGRDILEQELDIVSPEDLATPIGRLKVSLQAAAVLHAIYKEMTEDLFS **591 - 604:      KFTVVsdpLdeekK**  **>sp\|Q96KN7-3\|RPGR1_HUMAN** (645 aa) X-linked retinitis pigmentosa GTPase regulator-interacting protein 1 (RPGR-interacting protein 1) MLLMAPDRCRYVWKHCQPMEMRIKWIFLCCIRPLYDFTSQYVMETDSLFLHYLQEASARLDIHQAMASEHSTLAAGWICFDRVLETVEKVHGLATLIGAGGEEFGVLEYWMRLRFPIKPSLQACNKRKKAQVYLSTDVLGGRKAQEEEFRSESWEPQNELWIEITKCCGLRSRWLGTQPSPYAVYRFFTFSDHDTAIIPASNNPYFRDQARFPVLVTSDLDHYLRREALSIHVFDDEDLEPGSYLGRARVPLLPLAKNESIKGDFNLTDPAEKPNGSIQVQLDWKFPYIPPESFLKPEAQTKGKDTKDSSKISSEEEKASFPSQDQMASPEVPIEAGQYRSKRKPPHGGERKEKEHQVVSYSRRKHGKRIGVQGKNRMEYLSLNILNGNTPEQVNYTEWKFSETNSFIGDGFKNQHEEEEMTLSHSALKQKEPLHPVNDKESSEQGSEVSEAQTTDSDDVIVPPMSQKYPKADSEKMCIEIVSLAFYPEAEVMSDENIKQVYVEYKFYDLPLSETETPVSLRKPRAGEEIHFHFSKVIDLDPQEQQGRRRFLFDMLNGQDPDQGHLKFTVVSDPLDEEKKECEEVGYAYLQLWQILESGRDILEQELDIVSPEDLATPIGRLKVSLQAAAVLHAIYKEMTEDLFS **567 - 580:      KFTVVsdpLdeekK**  **>sp\|Q96KN7-4\|RPGR1_HUMAN** (612 aa) X-linked retinitis pigmentosa GTPase regulator-interacting protein 1 (RPGR-interacting protein 1) MLKLDNKDVISHPLGYPSESLLSIASMLDSSDSSSQPHWSNELIAEQLQQQVSQLQDQLDAELEDKRKVLLELSREKAQNEDLKLEVTNILQKHKQEVELLQNAATISQPPDRQSEPATHPAVLQENTQIEPSEPKNQEEKKLSQVLNELQVSHAETTLELEKTRDMLILQRKINVCYQEELEAMMTKADNDNRDHKEKLERLTRLLDLKNNRIKQLEGILRSHDLPTSGDFNLTDPAEKPNGSIQVQLDWKFPYIPPESFLKPEAQTKGKDTKDSSKISSEEEKASFPSQDQMASPEVPIEAGQYRSKRKPPHGGERKEKEHQVVSYSRRKHGKRIGVQGKNRMEYLSLNILNGNTPEQVNYTEWKFSETNSFIGDGFKNQHEEEEMTLSHSALKQKEPLHPVNDKESSEQGSEVSEAQTTDSDDVIVPPMSQKYPKADSEKMCIEIVSLAFYPEAEVMSDENIKQVYVEYKFYDLPLSETETPVSLRKPRAGEEIHFHFSKVIDLDPQEQQGRRRFLFDMLNGQDPDQGHLKFTVVSDPLDEEKKECEEVGYAYLQLWQILESGRDILEQELDIVSPEDLATPIGRLKVSLQAAAVLHAIYKEMTEDLFS **534 - 547:      KFTVVsdpLdeekK**  **>sp\|Q96KN7-5\|RPGR1_HUMAN** (902 aa) X-linked retinitis pigmentosa GTPase regulator-interacting protein 1 (RPGR-interacting protein 1) MLDSSDSSSQPHWSNELIAEQLQQQVSQLQDQLDAELEDKRKVLLELSREKAQNEDLKLEVTNILQKHKQEVELLQNAATISQPPDRQSEPATHPAVLQENTQIEPSEPKNQEEKKLSQVLNELQVSHAETTLELEKTRDMLILQRKINVCYQEELEAMMTKADNDNRDHKEKLERLTRLLDLKNNRIKQLEGILRSHDLPTSEQLKDVAYGTRPLSLCLETLPAHGDEDKVDISLLHQGENLFELHIHQAFLTSAALAQAGDTQPTTFCTYSFYDFETHCTPLSVGPQPLYDFTSQYVMETDSLFLHYLQEASARLDIHQAMASEHSTLAAGWICFDRVLETVEKVHGLATLIGAGGEEFGVLEYWMRLRFPIKPSLQACNKRKKAQVYLSTDVLGGRKAQEEEFRSESWEPQNELWIEITKCCGLRSRWLGTQPSPYAVYRFFTFSDHDTAIIPASNNPYFRDQARFPVLVTSDLDHYLRREALSIHVFDDEDLEPGSYLGRARVPLLPLAKNESIKGDFNLTDPAEKPNGSIQVQLDWKFPYIPPESFLKPEAQTKGKDTKDSSKISSEEEKASFPSQDQMASPEVPIEAGQYRSKRKPPHGGERKEKEHQVVSYSRRKHGKRIGVQGKNRMEYLSLNILNGNTPEQVNYTEWKFSETNSFIGDGFKNQHEEEEMTLSHSALKQKEPLHPVNDKESSEQGSEVSEAQTTDSDDVIVPPMSQKYPKADSEKMCIEIVSLAFYPEAEVMSDENIKQVYVEYKFYDLPLSETETPVSLRKPRAGEEIHFHFSKVIDLDPQEQQGRRRFLFDMLNGQDPDQGHLKFTVVSDPLDEEKKECEEVGYAYLQLWQILESGRDILEQELDIVSPEDLATPIGRLKVSLQAAAVLHAIYKEMTEDLFS **824 - 837:      KFTVVsdpLdeekK** |
| **>sp\|Q9HBT7\|ZN287_HUMAN** (761 aa) Zinc finger protein 287 (Zinc finger protein with KRAB and SCAN domains 13) MLASSKRMNSSSRSQILLRWKSDKAQSGPYNVEKEILTSRFLRDTETCRQNFRNFPYPDLAGPRKALSQLRELCLKWLRPEIHSKEQILELLVLEQFLTILPGEVRTWVKSQYPESSEEAVTLVEDLTQILEEEAPQNSTLSQDTPEEDPRGKHAFQTGWLNDLVTKESMTFKDVAVDITQEDWELMRPVQKELYKTVTLQNYWNMVSLGLTVYRPTVIPILEEPWMVIKEILEGPSPEWETKAQACTPVEDMSKLTKEETHTIKLEDSYDYDDRLERRGKGGFWKIHTDERGFSLKSVLSQEYDPTEECLSKYDIYRNNFEKHSNLIVQFDTQLDNKTSVYNEGRATFNHVSYGIVHRKILPGEKPYKCNVCGKKFRKYPSLLKHQSTHAKEKSYECEECGKEFRHISSLIAHQRMHTGEKPYECHQCGKAFSQRAHLTIHQRIHTGEKPYKCDDCGKDFSQRAHLTIHQRTHTGEKPYKCLECGKTFSHSSSLINHQRVHTGEKPYICNECGKTFSQSTHLLQHQKIHTGKKPYKCNECWKVFSQSTYLIRHQRIHSGEKCYKCNECGKAFAHSSTLIQHQTTHTGEKSYICNICGKAFSQSANLTQHHRTHTGEKPYKCSVCGKAFSQSVHLTQHQRIHNGEKPFKCNICGKAYRQGANLTQHQRIHTGEKPYKCNECGKAFIYSSSLNQHQRTHTGERPYKCNECDKDFSQRTCLIQHQRIHTGEKPYACRICGKTFTQSTNLIQHQRVHTGAKHRN **353 - 366:      SYGIVhrkIlpgeK** |

**Table S2.** Gene Ontology (GO) enrichment analysis of the proteins identified as putative GCs. Categories: biological process (BP), molecular function (MF), cellular component (CC). FDR – false discover rate.

| **Category** | **Term ID** | **Term description** | **FDR** |
| --- | --- | --- | --- |
| GO BP | GO:0009152 | Purine ribonucleotide biosynthetic process | 2.39e-12 |
| GO BP | GO:0007168 | Receptor guanylyl cyclase signaling pathway | 5.38e-10 |
| GO BP | GO:0006796 | Phosphate-containing compound metabolic process | 1.61e-6 |
| GO BP | GO:0019934 | cGMP-mediated signaling | 1.84e-6 |
| GO BP | GO:0035556 | Intracellular signal transduction | 3.14e-6 |
| GO BP | GO:0006468 | Protein phosphorylation | 0.00011 |
| GO BP | GO:0099555 | Trans-synaptic signaling by nitric oxide, modulating synaptic transmission | 0.0012 |
| GO BP | GO:0003008 | System process | 0.0018 |
| GO BP | GO:0044249 | Cellular biosynthetic process | 0.0019 |
| GO BP | GO:0019932 | Second-messenger-mediated signaling | 0.0021 |
| GO BP | GO:0006139 | Nucleobase-containing compound metabolic process | 0.0022 |
| GO BP | GO:1901576 | Organic substance biosynthetic process | 0.0027 |
| GO BP | GO:0010646 | Regulation of cell communication | 0.0030 |
| GO BP | GO:0023051 | Regulation of signaling | 0.0031 |
| GO BP | GO:0010750 | Positive regulation of nitric oxide mediated signal transduction | 0.0032 |
| GO BP | GO:0007167 | Enzyme linked receptor protein signaling pathway | 0.0035 |
| GO BP | GO:0010753 | Positive regulation of cGMP-mediated signaling | 0.0051 |
| GO BP | GO:1901564 | Organonitrogen compound metabolic process | 0.0166 |
| GO BP | GO:1902533 | Positive regulation of intracellular signal transduction | 0.0233 |
| GO BP | GO:0007263 | Nitric oxide mediated signal transduction | 0.0283 |
| GO BP | GO:0006807 | Nitrogen compound metabolic process | 0.0403 |
| GO BP | GO:0007166 | Cell surface receptor signaling pathway | 0.0408 |
| GO BP | GO:0008015 | Blood circulation | 0.0474 |
| GO BP | GO:0009966 | Regulation of signal transduction | 0.0474 |
| GO BP | GO:0022400 | Regulation of rhodopsin mediated signaling pathway | 0.0474 |
| GO MF | GO:0004383 | Guanylate cyclase activity | 9.98e-19 |
| GO MF | GO:0016941 | Natriuretic peptide receptor activity | 6.79e-11 |
| GO MF | GO:0005525 | GTP binding | 1.90e-9 |
| GO MF | GO:0017046 | Peptide hormone binding | 2.03e-7 |
| GO MF | GO:0035639 | Purine ribonucleoside triphosphate binding | 2.49e-6 |
| GO MF | GO:0032555 | Purine ribonucleotide binding | 3.38e-6 |
| GO MF | GO:0042277 | Peptide binding | 1.47e-5 |
| GO MF | GO:0004672 | Protein kinase activity | 2.22e-5 |
| GO MF | GO:0038023 | Signaling receptor activity | 0.00074 |
| GO MF | GO:0005524 | ATP binding | 0.0082 |
| GO MF | GO:0020037 | Heme binding | 0.0158 |
| GO MF | GO:1901363 | Heterocyclic compound binding | 0.0213 |
| GO MF | GO:0097159 | Organic cyclic compound binding | 0.0241 |
| GO MF | GO:0043167 | Ion binding | 0.0375 |
| GO CC | GO:0008074 | Guanylate cyclase complex, soluble | 0.0064 |
|  |  |  |  |

**Table S3.** Domain enrichment analysis of the proteins identified as putative GCs. Protein domains were retrieved from Pfam, InterPro, and SMART database. FDR – false discover rate.

| **Category** | **Term ID** | **Term description** | **FDR** |
| --- | --- | --- | --- |
| Pfam | PF00211 | Adenylate and Guanylate cyclase catalytic domain | 1.29e-16 |
| Pfam | PF07701 | Heme NO binding associated | 7.89e-11 |
| Pfam | PF07700 | Haem-NO-binding | 1.03e-5 |
| Pfam | PF01094 | Receptor family ligand binding region | 2.30e-5 |
| Pfam | PF00069 | Protein kinase domain | 3.71e-5 |
| Pfam | PF07714 | Protein tyrosine kinase | 3.71e-5 |
| InterPro | IPR001054 | Adenylyl cyclase class-3/4/guanylyl cyclase | 1.75e-16 |
| InterPro | IPR018297 | Adenylyl cyclase class-4/guanylyl cyclase, conserved site | 1.75e-16 |
| InterPro | IPR029787 | Nucleotide cyclase | 1.75e-16 |
| InterPro | IPR011645 | Haem NO binding associated | 7.75e-11 |
| InterPro | IPR001828 | Receptor, ligand binding region | 2.06e-7 |
| InterPro | IPR028082 | Periplasmic binding protein-like I | 2.19e-7 |
| InterPro | IPR001245 | Serine-threonine/tyrosine-protein kinase, catalytic domain | 8.03e-7 |
| InterPro | IPR011644 | Heme NO-binding | 7.56e-6 |
| InterPro | IPR038158 | H-NOX domain superfamily | 7.56e-6 |
| InterPro | IPR042463 | Haem NO binding associated domain superfamily | 7.56e-6 |
| InterPro | IPR000719 | Protein kinase domain | 3.41e-5 |
| InterPro | IPR024096 | NO signalling/Golgi transport ligand-binding domain superfamily | 4.15e-5 |
| InterPro | IPR011009 | Protein kinase-like domain superfamily | 5.26e-5 |
| InterPro | IPR001170 | Adenylyl cyclase class-4/guanylyl cyclase | 0.0030 |
| SMART | SM00044 | Adenylyl- / guanylyl cyclase, catalytic domain | 5.50e-12 |
|  |  |  |  |

**Table S4.** Pathway enrichment analysis of the proteins identified as putative GCs are involved in. Pathways were retrieved from KEGG, Reactome, and WikiPathways database. FDR – false discover rate; WikiPs – WikiPathways.

| **Category** | **Term ID** | **Term description** | **FDR** |
| --- | --- | --- | --- |
| KEGG | hsa00230 | Purine metabolism | 1.71e-11 |
| KEGG | hsa04270 | Vascular smooth muscle contraction | 1.11e-5 |
| KEGG | hsa04921 | Oxytocin signaling pathway | 1.29e-5 |
| KEGG | hsa04022 | cGMP-PKG signaling pathway | 1.44e-5 |
| KEGG | hsa04924 | Renin secretion | 1.77e-5 |
| KEGG | hsa01100 | Metabolic pathways | 0.00038 |
| KEGG | hsa04730 | Long-term depression | 0.00079 |
| KEGG | hsa04540 | Gap junction | 0.0021 |
| KEGG | hsa04713 | Circadian entrainment | 0.0021 |
| KEGG | hsa04970 | Salivary secretion | 0.0021 |
| KEGG | hsa04611 | Platelet activation | 0.0041 |
| KEGG | hsa04744 | Phototransduction | 0.0065 |
| Reactome | HSA-397014 | Muscle contraction | 0.00087 |
| Reactome | HSA-392154 | Nitric oxide stimulates guanylate cyclase | 0.0011 |
| Reactome | HSA-445355 | Smooth Muscle Contraction | 0.0034 |
| Reactome | HSA-5578768 | Physiological factors | 0.0264 |
| WikiPs | WP4008 | NO/cGMP/PKG mediated neuroprotection | 4.54e-5 |
| WikiPs | WP5113 | Antiviral and anti-inflammatory effects of Nrf2 on SARS-CoV-2 pathway | 0.00089 |
| WikiPs | WP4222 | Phosphodiesterases in neuronal function | 0.0024 |
|  |  |  |  |

**Table S5.** Functional enrichment analysis in the local network cluster, disease-gene associations, and subcellular localization of the proteins identified as putative GCs. Terms were retrieved from STRING clusters, DISEASES, and COMPARTMENTS database, while annotated keywords were retrieved from UniProt. FDR – false discover rate; COMPs – COMPARTMENTS.

| **Category** | **Term ID** | **Term description** | **FDR** |
| --- | --- | --- | --- |
| STRING | CL:24025 | cGMP metabolic process, and cGMP-dependent kinase | 2.76e-8 |
| STRING | CL:24028 | Heme NO-binding, and cGMP-dependent kinase | 5.68e-5 |
| STRING | CL:24085 | Phototransduction, and Cone-rod dystrophy | 0.0052 |
| STRING | CL:24103 | Leber congenital amaurosis 1, and Guanylyl cyclase-activating protein 3 | 0.0074 |
| STRING | CL:24048 | Natriuretic peptide, and Adenylyl cyclase class-4/guanylyl cyclase | 0.0079 |
| DISEASES | DOID:14791 | Leber congenital amaurosis | 0.0105 |
| DISEASES | DOID:0110078 | Leber congenital amaurosis 1 | 0.0205 |
| COMPs | GOCC:0008074 | Guanylate cyclase complex, soluble | 0.0404 |
| COMPs | GOCC:0097733 | Photoreceptor cell cilium | 0.0404 |
| COMPs | GOCC:0005640 | Nuclear outer membrane | 0.0487 |
| UniProt | KW-0141 | cGMP biosynthesis | 2.09e-19 |
| UniProt | KW-0456 | Lyase | 8.80e-11 |
| UniProt | KW-0342 | GTP-binding | 3.50e-10 |
| UniProt | KW-0547 | Nucleotide-binding | 1.55e-6 |
| UniProt | KW-0844 | Vision | 0.0153 |
| UniProt | KW-0182 | Cone-rod dystrophy | 0.0263 |
| UniProt | KW-0901 | Leber congenital amaurosis | 0.0263 |
|  |  |  |  |

**Table S6.** List of primers used in cloning NTRK1 constructs.

| **Primer name** | **Sequence 5’ – 3’** * |
| --- | --- |
| NTRK1cd GW FP | GGGGACAAGTTTGTACAAAAAAGCAGGCTTCGAAGGAGATAGAACCATGGggATGAAATGTGGACGGAGAAAC |
| NTKR1 stop GW RP | GGGGACCACTTTGTACAAGAAAGCTGGGTCCTAGCCCAGGACATCCAGGTA |

* Gateway sequence in green, Shine-Dalgarno and Kozak sequence in pink, NTRK1 specific sequence in blue, Start and Stop in red.

NTRK1-KD MSLHFMTLGGSSLSPTEGKGSGLQGHIIENPQYFSDACVHHIKRRDIVLKWELGEGAFGK 60

PSKR1-KD ------------------------DDLLDSTNSFD-------------QANIIGCGGFGM 23

.:::. : *. :* *.**

NTRK1-KD VFLAECHNLLPEQDKMLVAVKALKEASESARQDFQREAELLTMLQHQHIVRFFGVCTEGR 120

PSKR1-KD VYKAT----LPDG--KKVAIKKLSGDCGQIEREFEAEVETLSRAQHPNLVLLRGFCFYKN 87

*: * **: **:* *. . . .::*: *.* *: ** .:* : *.* .

NTRK1-KD PLLMVFEYMRHGDLNRFLRSHGPDAKLLAGGEDVAPGPLGLGQLLAVASQVAAGMVYLA- 179

PSKR1-KD DRLLIYSYMENGSLDYWLHERNDGPALLK-----------WKTRLRIAQGAAKGLLYLHE 136

*:::.**..*.*: :*:.: ** * :*. .* *::**

NTRK1-KD --GLHFVHRDLATRNCLVGQGLVVKIGDFGMSRDIYSTDYYRVGGRTMLPIRWMPPESIL 237

PSKR1-KD GCDPHILHRDIKSSNILLDENFNSHLADFGLARLMSPYE-THVSTDLVGTLGYIPPEYGQ 195

*::***: : * *: : : ::.***::* : : :*. : : ::***

NTRK1-KD YRKFTTESDVWSFGVVLWEIFTYGKQPWYQLSNTEAIDCIT---QG-RELERPRACPPE- 292

PSKR1-KD ASVATYKGDVYSFGVVLLELLTDKRPVDM-CKPKGCRDLISWVVKMKHESRASEVFDPLI 254

* :.**:****** *::* : . . . * *: : :* . .. *

NTRK1-KD -----------VYAIMRGCWQREPQQRHSIKDVHARLQALAQAPPVYLDVLG 333

PSKR1-KD YSKENDKEMFRVLEIACLCLSENPKQRPTTQQLVSWLDDV------------ 284

* * * ..:*:** : ::: : *: :

**Figure S1**. Alignment of the kinase domain of NTRK1 and PSKR1 used for homology modelling. Alignment generated by Clustal Omega. The G-loop is highlighted in yellow, VAIK in grey, the hinge in olive, HRD in cyan, DFG in pink, activation segment in red and the GC catalytic centre in green while the tyrosine kinase catalytic domain of NTRK1 is underlined. Identical residues are indicated with an asterisk (*), conserved residues with a colon (:), and semi-conserved residues with a full stop (.).

**A. NTRK1 orthologues**

****:******** *********:*****::  ****:*****: *:*******

ref|XP_020653829.1| MSRDIYSTDYYRVGGRTMLPIRWMPPESILYRKFTTESDIWSFGVVLWEIFTYGKQPWYQ 750

ref|XP_016159478.1| MSRDIYSTDYYRVGGRTMLPIRWMPPESILYRKFTTESDIWSFGVVLWEIFTYGKQPWYQ 744

ref|XP_025902407.1| MSRDIYSTDYYRVGGRTMLPIRWMPPESILYRKFTAESDIWSFGVVLWEIFTYGKQPWYQ 669

ref|XP_026654962.1| MSRDIYSTDYYRVGGRTMLPIRWMPPESILYRKFTTESDIWSFGVVLWEIFTYGKQPWYQ 695

ref|XP_021387944.1| MSRDIYSTDYYRVGGRTMLPIRWMPPESILYRKFTTESDVWSFGVVLWEIFTYGKQPWYQ 690

ref|XP_036253615.1| MSRDIYSTDYYRVGGRTMLPIRWMPPESILYRKFTTESDIWSFGVVLWEIFTYGKQPWYQ 728

ref|XP_014166220.1| MSRDIYSTDYYRVGGRTMLPIRWMPPESILYRKFTTESDIWSFGVVLWEIFTYGKQPWYQ 674

ref|XP_030821172.1| MSRDIYSTDYYRVGGRTMLPIRWMPPESILYRKFTTESDIWSFGVVLWEIFTYGKQPWYQ 731

ref|XP_041576946.1| MSRDIYSTDYYRVGGRTMLPIRWMPPESILYRKFTTESDIWSFGVVLWEIFTYGKQPWYQ 730

ref|XP_039558220.1| MSRDIYSTDYYRVGGRTMLPIRWMPPESILYRKFTTESDIWSFGVVLWEIFTYGKQPWYQ 725

ref|XP_041253220.1| MSRDIYSTDYYRVGGRTMLPIRWMPPESILYRKFTTESDIWSFGVVLWEIFTYGKQPWYQ 728

ref|XP_041320820.1| MSRDIYSTDYYRVGGRTMLPIRWMPPESILYRKFTTESDIWSFGVVLWEIFTYGKQPWYQ 728

ref|XP_030304931.1| MSRDIYSTDYYRVGAGPMLPIRWMPPESILYRKFTTESDIWSFGVVLWEIFTYGKQPWYQ 722

ref|XP_027654888.1| MSRDIYSTDYYRVGGRTMLPIRWMPPESILYRKFTTESDIWSFGVVLWEIFTYGKQPWYQ 708

ref|XP_021232487.1| MSRDIYSTDYYRVGGRTMLPIRWMPPESILYRKFTTESDIWSFGVVLWEIFTYGKQPWYQ 725

ref|XP_015739850.1| MSRDIYSTDYYRVGGHTMLPIRWMPPESILYRKFTTESDIWSFGVVLWEIFTYGKQPWYQ 719

ref|NP_990709.1| MSRDIYSTDYYRVGGRTMLPIRWMPPESILYRKFTTESDIWSFGVVLWEIFTYGKQPWYQ 724

ref|XP_031412765.1| MSRDIYSTDYYRVGGRTMLPIRWMPPESILYRKFTTESDIWSFGVVLWEIFTYGKQPWYQ 702

ref|XP_031462179.1| MSRDIYSTDYYRVGGRTMLPIRWMPPESILYRKFTTESDIWSFGVVLWEIFTYGKQPWYQ 724

ref|XP_032060533.1| MSRDIYSTDYYRVGGRTMLPIRWMPPESILYRKFTTESDIWSFGVVLWEIFTYGKQPWYQ 730

ref|XP_035168830.1| MSRDIYSTDYYRVGGRTMLPIRWMPPESILYRKFTTESDIWSFGVVLWEIFTYGKQPWYQ 671

ref|XP_035423379.1| MSRDIYSTDYYRVGGRTMLPIRWMPPESILYRKFTTESDIWSFGVVLWEIFTYGKQPWYQ 728

ref|XP_040394524.1| MSRDIYSTDYYRVGGRTMLPIRWMPPESILYRKFTTESDIWSFGVVLWEIFTYGKQPWYQ 722

ref|XP_025929628.1| MSRDIYSTDYYRVGGRTMLPIRWMPPESILYRKFTTESDIWSFGVVLWEIFTYGKQPWYQ 728

ref|XP_033927202.1| MSRDIYSTDYYRVGGRTMLPIRWMPPESILYRKFTTESDIWSFGVVLWEIFTYGKQPWYQ 727

ref|XP_030330060.1| MSRDIYSTDYYRVGAGPCCPIRWMPPESILYRKFTTESDIWSFGVVLWEIFTYGKQPWYQ 727

ref|XP_037230679.1| MSRDIYSTDYYRVGGRTMLPIRWMPPESILYRKFTTESDIWSFGVVLWEIFTYGKQPWYQ 730

ref|XP_040433952.1| MSRDIYSTDYYRVGGRTMLPIRWMPPESILYRKFTTESDIWSFGVVLWEIFTYGKQPWYQ 730

ref|XP_009465981.1| MSRDIYSTDYYRVGGRTMLPIRWMPPESILYRKFTTESDIWSFGVVLWEIFTYGKQPWYQ 664

ref|XP_010562499.1| MSRDIYSTDYYRVGGRTMLPIRWMPPESILYRKFTTESDIWSFGVVLWEIFTYGKQPWYQ 670

ref|XP_029879907.1| MSRDIYSTDYYRVGGRTMLPIRWMPPESILYRKFTTESDIWSFGVVLWEIFTYGKQPWYQ 751

ref|XP_027765205.1| MSRDIYSTDYYRVGGRTMLPIRWMPPESILYRKFTTESDIWSFGVVLWEIFTYGKQPWYQ 725

ref|XP_027554939.1| MSRDIYSTDYYRVGGRTMLPIRWMPPESILYRKFTTESDIWSFGVVLWEIFTYGKQPWYQ 781

ref|XP_017663883.1| MSRDIYSTDYYRVGGRTMLPIRWMPPESILYRKFTTESDIWSFGVVLWEIFTYGKQPWYQ 725

ref|XP_027598296.1| MSRDIYSTDYYRVGGRTMLPIRWMPPESILYRKFTTESDIWSFGVVLWEIFTYGKQPWYQ 725

ref|XP_027523545.1| MSRDIYSTDYYRVGGRTMLPIRWMPPESILYRKFTTESDIWSFGVVLWEIFTYGKQPWYQ 780

ref|XP_032568921.1| MSRDIYSTDYYRVGGRTMLPIRWMPPESILYRKFTTESDIWSFGVVLWEIFTYGKQPWYQ 725

ref|XP_017583342.1| MSRDIYSTDYYRVGGRTMLPIRWMPPESILYRKFTTESDIWSFGVVLWEIFTYGKQPWYQ 707

ref|XP_031949612.1| MSRDIYSTDYYRVGGRTMLPIRWMPPESILYRKFTTESDIWSFGVVLWEIFTYGKQPWYQ 726

ref|XP_041890545.1| MSRDIYSTDYYRVGGRTMLPIRWMPPESILYRKFTTESDIWSFGVVLWEIFTYGKQPWYQ 726

ref|XP_005532420.1| MSRDIYSTDYYRVGGRTMLPIRWMPPESILYRKFTTESDIWSFGVVLWEIFTYGKQPWYQ 727

ref|XP_015505499.1| MSRDIYSTDYYRVGGRTMLPIRWMPPESILYRKFTTESDIWSFGVVLWEIFTYGKQPWYQ 729

ref|XP_014744807.1| MSRDIYSTDYYRVGGRTMLPIRWMPPESILYRKFTTESDIWSFGVVLWEIFTYGKQPWYQ 727

ref|XP_032938729.1| MSRDIYSTDYYRVGGRTMLPIRWMPPESILYRKFTTESDIWSFGVVLWEIFTYGKQPWYQ 727

ref|XP_029437670.1| MSRDIYSTDYYRVGGRTMLPIRWMPPESILYRKFTTESDIWSFGVVLWEIFTYGKQPWYQ 724

ref|XP_030044091.1| MSRDIYSTDYYRVGGRTMLPIRWMPPESILYRKFTTESDIWSFGVVLWEIFTYGKQPWYQ 729

ref|XP_033779018.1| MSRDIYSTDYYRVGGRTMLPIRWMPPESILYRKFTTESDIWSFGVVLWEIFTYGKQPWYQ 718

ref|XP_002939035.2| MSRDIYSTDYYRVGGRTMLPIRWMPPESILYRKFTTESDIWSFGVVLWEIFTYGKQPWYQ 742

ref|XP_040267036.1| MSRDIYSTDYYRVGGRTMLPIRWMPPESILYRKFTTDSDIWSFGVVLWEIFTYGKQPWYQ 746

ref|XP_018429550.1| MSRDIYSTDYYRVGGRTMLPIRWMPPESILYRKFTTDSDIWSFGVVLWEIFTYGKQPWYQ 713

ref|XP_040189012.1| MSRDIYSTDYYRVGGRTMLPIRWMPPESILYRKFTTESDIWSFGVVLWEIFTCGKQPWYQ 727

ref|XP_025070144.1| MSRDIYSTDYYRVGGRTMLPIRWMPPESILYRKFTTESDIWSFGVVLWEIFTYGKQPWYQ 750

ref|XP_019351184.1| MSRDIYSTDYYRVGGRTMLPIRWMPPESILYRKFTTESDIWSFGVVLWEIFTYGKQPWYQ 750

ref|XP_019374557.1| MSRDIYSTDYYRVGGRTMLPIRWMPPESILYRKFTTXXXXXXXGVVLWEIFTYGKQPWYQ 750

ref|XP_019411321.1| MSRDIYSTDYYRVGGRTMLPIRWMPPESILYRKFTTESDIWSFGVVLWEIFTYGKQPWYQ 750

ref|XP_014424462.1| MSRDIYSTDYYRVGGRTMLPIRWMPPESILYRKFTAESDIWSFGVVLWEIFTYGKQPWYQ 696

ref|XP_032648119.1| MSRDIYSTDYYRVGGRTMLPIRWMPPESILYRKFTAESDIWSFGVVLWEIFTYGKQPWYQ 731

ref|XP_029768322.1| MSRDIYSTDYYRVGGRTMLPIRWMPPESILYRKFTAESDIWSFGVVLWEIFTYGKQPWYQ 706

ref|XP_005311302.2| MSRDIYSTDYYRVGGRTMLPIRWMPPESILYRKFTTESDIWSFGVVLWEIFTYGKQPWYQ 726

ref|XP_034612853.1| MSRDIYSTDYYRVGGRTMLPIRWMPPESILYRKFTTESDIWSFGVVLWEIFTYGKQPWYQ 697

ref|XP_030397932.1| MSRDIYSTDYYRVGGRTMLPIRWMPPESILYRKFTAESDIWSFGVVLWEIFTYGKQPWYQ 726

ref|XP_039369376.1| MSRDIYSTDYYRVGGRTMLPIRWMPPESILYRKFTAESDIWSFGVVLWEIFTYGKQPWYQ 732

ref|XP_037738825.1| MSRDIYSTDYYRVGGRTMLPIRWMPPESILYRKFTAESDIWSFGVVLWEIFTYGKQPWYQ 726

ref|XP_038238775.1| MSRDIYSTDYYRVGGRTMLPIRWMPPESILYRKFTAESDIWSFGVVLWEIFTYGKQPWYQ 726

ref|XP_003228453.1| MSRDIYSTDYYRVGGRTMLPIRWMPPESILYRKFTTESDIWSFGVVLWEIFTYGKQPWYQ 739

ref|XP_015271976.1| MSRDIYSTDYYRVGGRTMLPIRWMPPESILYRKFTTESDIWSFGVVLWEIFTYGKQPWYQ 673

ref|XP_033030856.1| MSRDIYSTDYYRVGGRTMLPIRWMPPESILYRKFTTESDIWSFGVVLWEIFTYGKQPWYQ 738

ref|XP_028566168.1| MSRDIYSTDYYRVGGRTMLPIRWMPPESILYRKFTTESDIWSFGVVLWEIFTYGKQPWYQ 792

ref|XP_034991029.1| MSRDIYSTDYYRVGGRTMLPIRWMPPESILYRKFTTESDIWSFGVVLWEIFTYGKQPWYQ 738

ref|XP_025028477.1| MSRDIYSTDYYRVGGRTMLPIRWMPPESILYRKFTTESDIWSFGVVLWEIFTYGKQPWYQ 526

ref|XP_015683378.1| MSRDIYSTDYYRVGGRTMLPIRWMPPESILYRKFTTESDIWSFGVVLWEIFTYGKQPWYQ 735

ref|XP_034280922.1| MSRDVYSTDYYRVGGRTMLPIRWMPPESILYRKFTTESDIWSFGVVLWEIFTYGKQPWYQ 736

ref|XP_026541704.1| MSRDVYSTDYYRVGGRTMLPIRWMPPESILYRKFTTESDIWSFGVVLWEIFTYGKQPWYQ 725

ref|XP_026575957.1| MSRDVYSTDYYRVGGRTMLPIRWMPPESILYRKFTTESDIWSFGVVLWEIFTYGKQPWYQ 725

ref|XP_016285031.1| MSRDIYSTDYYRVGGRTMLPIRWMPPESILYRKFTTESDVWSFGVLLWEIFTYGKQPWYQ 650

ref|XP_031822658.1| MSRDIYSTDYYRVGGRTMLPIRWMPPESILYRKFTTESDVWSFGVLLWEIFTYGKQPWYQ 729

ref|XP_036610432.1| MSRDIYSTDYYRVGGRTMLPIRWMPPESILYRKFTTESDVWSFGVLLWEIFTYGKQPWYQ 729

ref|XP_020847636.1| MSRDIYSTDYYRVGGRTMLPIRWMPPESILYRKFTTESDVWSFGVLLWEIFTYGKQPWYQ 729

ref|XP_027727094.1| MSRDIYSTDYYRVGGRTMLPIRWMPPESILYRKFTTESDVWSFGVLLWEIFTYGKQPWYQ 729

ref|XP_012885969.1| MSRDIYSTDYYRVGGRTMLPIRWMPPESILYRKFTTESDVWSFGVVLWEIFTYGKQPWYQ 688

ref|XP_020018986.1| MSRDIYSTDYYRVGGRTMLPIRWMPPESILYRKFTTESDVWSFGVVLWEIFTYGKQPWYQ 752

ref|XP_012806314.1| MSRDIYSTDYYRVGGRTMLPIRWMPPESILYRKFSTESDVWSFGVVLWEIFTYGKQPWYQ 708

ref|XP_008851531.1| MSRDIYSTDYYRVGGRTMLPIRWMPPESILYRKFSTESDVWSFGVVLWEIFTYGKQPWYQ 732

ref|XP_021518216.1| MSRDIYSTDYYRVGGRTMLPIRWMPPESILYRKFSTESDVWSFGVVLWEIFTYGKQPWYQ 733

ref|XP_005080156.1| MSRDIYSTDYYRVGGRTMLPIRWMPPESILYRKFSTESDVWSFGVVLWEIFTYGKQPWYQ 733

ref|XP_028608940.1| MSRDIYSTDYYRVGGRTMLPIRWMPPESILYRKFSTESDVWSFGVVLWEIFTYGKQPWYQ 733

ref|XP_034356559.1| MSRDIYSTDYYRVGGRTMLPIRWMPPESILYRKFSTESDVWSFGVVLWEIFTYGKQPWYQ 733

ref|NP_067600.1| MSRDIYSTDYYRVGGRTMLPIRWMPPESILYRKFSTESDVWSFGVVLWEIFTYGKQPWYQ 733

ref|XP_032754025.1| MSRDIYSTDYYRVGGRTMLPIRWMPPESILYRKFSTESDVWSFGVVLWEIFTYGKQPWYQ 733

ref|XP_021051015.1| MSRDIYSTDYYRVGGRTMLPIRWMPPESILYRKFSTESDVWSFGVVLWEIFTYGKQPWYQ 733

ref|NP_001028296.1| MSRDIYSTDYYRVGGRTMLPIRWMPPESILYRKFSTESDVWSFGVVLWEIFTYGKQPWYQ 733

ref|XP_021013390.1| MSRDIYSTDYYRVGGRTMLPIRWMPPESILYRKFSTESDVWSFGVVLWEIFTYGKQPWYQ 733

ref|XP_003502945.1| MSRDIYSTDYYRVGGRTMLPIRWMPPESILYRKFSTESDVWSFGVVLWEIFTYGKQPWYQ 733

ref|XP_036046383.1| MSRDIYSTDYYRVGGRTMLPIRWMPPESILYRKFSTESDVWSFGVVLWEIFTYGKQPWYQ 733

ref|XP_006976478.1| MSRDIYSTDYYRVGGRTMLPIRWMPPESILYRKFSTESDVWSFGVVLWEIFTYGKQPWYQ 707

ref|XP_028713494.1| MSRDIYSTDYYRVGGRTMLPIRWMPPESILYRKFSTESDVWSFGVVLWEIFTYGKQPWYQ 733

ref|XP_005356966.1| MSRDIYSTDYYRVGGRTMLPIRWMPPESILYRKFSTESDVWSFGVVLWEIFTYGKQPWYQ 733

ref|XP_041511939.1| MSRDIYSTDYYRVGGRTMLPIRWMPPESILYRKFSTESDVWSFGVVLWEIFTYGKQPWYQ 733

ref|XP_038167132.1| MSRDIYSTDYYRVGGRTMLPIRWMPPESILYRKFSTESDVWSFGVVLWEIFTYGKQPWYQ 733

ref|XP_019066142.1| MSRDIYSTDYYRVGGRTMLPIRWMPPESILYRKFTTESDVWSFGVVLWEIFTYGQQPWYQ 727

ref|XP_004871305.1| MSRDIYSTDYYRVGGRTMLPIRWMPPESILYRKFTTESDVWSFGVVLWEIFTYGQQPWYQ 732

ref|XP_003475689.1| MSRDIYSTDYYRVGGRTMLPIRWMPPESILYRKFTAESDVWSFGVVLWEIFTYGQQPWYQ 732

ref|XP_004629777.1| MSRDIYSTDYYRVGGRTMLPIRWMPPESILYRKFTAESDVWSFGVVLWEIFTYGQQPWYQ 732

ref|XP_005411770.1| MSRDIYSTDYYRVGGRTMLPIRWMPPESILYRKFTAESDVWSFGVVLWEIFTYGQQPWYQ 742

ref|XP_004778692.1| MSRDIYSTDYYRVGGRTMLPIRWMPPESLLYRKFTTESDVWSFGVVLWEIFTYGKQPWYQ 698

ref|XP_029791805.1| MSRDIYSTDYYRVGGRTMLPIRWMPPESILYRKFTTESDVWSFGVVLWEIFTYGKQPWYQ 704

ref|XP_003415212.1| MSRDIYSTDYYRVGGRTMLPIRWMPPESILYRKFTTESDVWSFGVVLWEIFTYGKQPWYQ 725

ref|XP_032947886.1| MSRDIYSTDYYRVGGRTMLPIRWMPPESILYRKFTTESDVWSFGVLLWEIFTYGKQPWYQ 730

ref|XP_036298488.1| MSRDIYSTDYYRVGGRTMLPIRWMPPESILYRKFTTESDVWSFGVLLWEIFTFGKQPWYQ 728

ref|XP_016049105.1| MSRDIYSTDYYRVGGRTMLPIRWMPPESILYRKFTTESDVWSFGVVLWEIFTYGKQPWYQ 670

ref|XP_008262512.1| MSRDIYSTDYYRVGGRTMLPIRWMPPESILYRKFSTESDVWSFGVVLWEIFTYGKQPWYQ 731

ref|XP_012783524.1| MSRDIYSTDYYRVGGRTMLPIRWMPPESILYRKFTTESDVWSFGVVLWEIFTYGKQPWYQ 692

ref|XP_040844446.1| MSRDIYSTDYYRVGGRTMLPIRWMPPESILYRKFTTESDVWSFGVVLWEIFTYGKQPWYQ 729

ref|XP_004480634.1| MSRDIYSTDYYRVGGRTMLPIRWMPPESILYRKFTTESDVWSFGVVLWEIFTYGKQPWYQ 735

ref|XP_027630285.1| MSRDIYSTDYYRVXXRPIRPIRWMPPESILYRKFTTESDVWSFGVVLWEIFTYGKQPWYQ 691

ref|XP_008153851.1| MSRDIYSTDYYRVGGRTMLPIRWMPPESILYRKFTTESDVWSFGVLLWEIFTFGKQPWYQ 732

ref|XP_036200359.1| MSRDIYSTDYYRVGGRTMLPIRWMPPESILYRKFTTESDVWSFGVLLWEIFTYGKQPWYQ 728

ref|XP_016076811.1| MSRDIYSTDYYRVGGRTMLPIRWMPPESILYRKFTTESDVWSFGVLLWEIFTYGKQPWYQ 725

ref|XP_036128147.1| MSRDIYSTDYYRVGGRTMLPIRWMPPESILYRKFTTESDVWSFGVLLWEIFTYGKQPWYQ 734

ref|XP_024428203.1| MSRDIYSTDYYRVRGHTMLPIRWMPPESILYRKFTTESDVWSFGVLLWEIFTYGKQPWYQ 710

ref|XP_028358794.1| MSRDIYSTDYYRVGGRTMLPIRWMPPESILYRKFTTESDVWSFGVLLWEIFTYGKQPWYQ 730

ref|XP_036988443.1| MSRDIYSTDYYRVGGRTMLPIRWMPPESILYRKFTTESDVWSFGVLLWEIFTYGKQPWYQ 730

ref|XP_007172080.1| MSRDIYSTDYYRVGGRTMLPIRWMPPESILYRKFTTESDVWSFGVVLWEIFTYGKQPWYQ 760

ref|XP_006861670.1| MSRDIYSTDYYRVGGRTMLPIRWMPPESILYRKFTTESDVWSFGVVLWEIFTYGKQPWYQ 730

ref|XP_006895804.1| MSRDIYSTDYYRVGGRTMLPIRWMPPESILYRKFTTESDVWSFGVVLWEIFTYGKQPWYQ 750

ref|XP_015358198.1| MSRDIYSTDYYRVGGRTMLPIRWMPPESILYRKFTTESDVWSFGVVLWEIFTYGKQPWYQ 707

ref|XP_027776798.1| MSRDIYSTDYYRVGGRTMLPIRWMPPESILYRKFTTESDVWSFGVVLWEIFTYGKQPWYQ 733

ref|XP_005331464.1| MSRDIYSTDYYRVGGRTMLPIRWMPPESILYRKFTTESDVWSFGVVLWEIFTYGKQPWYQ 733

ref|XP_026260808.1| MSRDIYSTDYYRVGGRTMLPIRWMPPESILYRKFTTESDVWSFGVVLWEIFTYGKQPWYQ 733

ref|XP_023598371.1| MSRDIYSTDYYRVGGRTMLPIRWMPPESILYRKFTTESDVWSFGVVLWEIFTYGKQPWYQ 704

ref|XP_007946609.1| MSRDIYSTDYYRVGGRTMLPIRWMPPESILYRKFTTESDVWSFGVVLWEIFTYGKQPWYQ 728

ref|XP_001929560.2| MSRDIYSTDYYRVGGRTMLPIRWMPPESILYRKFTTESDVWSFGVVLWEIFTYGKQPWYQ 729

ref|XP_016021682.2| MSRDIYSTDYYRVGGRTMLPIRWMPPESILYRKFTTESDVWSFGVLLWEIFAYGKQPWYQ 729

ref|XP_039723191.1| MSRDIYSTDYYRVGGRTMLPIRWMPPESILYRKFTTESDVWSFGVLLWEIFAYGKQPWYQ 729

ref|XP_003795284.1| MSRDIYSTDYYRVGGRTMLPIRWMPPESILYRKFTAESDVWSFGVVLWEIFTYGKQPWYQ 730

ref|XP_008568499.1| MSRDIYSTDYYRVGGRTMLPIRWMPPESILYRKFTTESDVWSFGVVLWEIFTYGKQPWYQ 753

ref|XP_004693915.1| MSRDIYSTDYYRVGGRTMLPIRWMPPESILYRKFTAESDVWSFGVVLWEIFTYGKQPWYQ 729

ref|XP_037355985.1| MSRDIYSTDYYRVGGRTMLPIRWMPPESILYRKFTTESDVWSFGVVLWEIFTYGKQPWYQ 729

ref|XP_025852701.1| MSRDIYSTDYYRVGGRTMLPIRWMPPESILYRKFTTESDVWSFGVVLWEIFTYGKQPWYQ 718

ref|XP_038426254.1| MSRDIYSTDYYRVGGRTMLPIRWMPPESILYRKFTTESDVWSFGVVLWEIFTYGKQPWYQ 730

ref|XP_025286580.1| MSRDIYSTDYYRVGGRTMLPIRWMPPESILYRKFTTESDVWSFGVVLWEIFTYGKQPWYQ 730

ref|XP_041598713.1| MSRDIYSTDYYRVGGRTMLPIRWMPPESILYRKFTTESDVWSFGVVLWEIFTYGKQPWYQ 729

ref|XP_032700958.1| MSRDIYSTDYYRVGGRTMLPIRWMPPESLLYRKFTTESDVWSFGVVLWEIFTYGKQPWYQ 730

ref|XP_022346513.1| MSRDIYSTDYYRVGGRTMLPIRWMPPESLLYRKFTTESDVWSFGVVLWEIFTYGKQPWYQ 730

ref|XP_032173974.1| MSRDIYSTDYYRVGGRTMLPIRWMPPESLLYRKFTTESDVWSFGVVLWEIFTYGKQPWYQ 730

ref|XP_026340884.1| MSRDIYSTDYYRVGGRTMLPIRWMPPESILYRKFTTESDVWSFGVVLWEIFTYGKQPWYQ 730

ref|XP_004402946.1| MSRDIYSTDYYRVGGRTMLPIRWMPPESILYRKFTTESDVWSFGVVLWEIFTYGKQPWYQ 730

ref|XP_025714628.1| MSRDIYSTDYYRVGGRTMLPIRWMPPESILYRKFTTESDVWSFGVVLWEIFTYGKQPWYQ 730

ref|XP_027467848.2| MSRDIYSTDYYRVGGRTMLPIRWMPPESILYRKFTTESDVWSFGVVLWEIFTYGKQPWYQ 730

ref|XP_027945997.1| MSRDIYSTDYYRVGGRTMLPIRWMPPESILYRKFTTESDVWSFGVVLWEIFTYGKQPWYQ 730

ref|XP_034865733.1| MSRDIYSTDYYRVGGRTMLPIRWMPPESILYRKFTTESDVWSFGVVLWEIFTYGKQPWYQ 724

ref|XP_032254228.1| MSRDIYSTDYYRVGGRTMLPIRWMPPESILYRKFTTESDVWSFGVVLWEIFTYGKQPWYQ 730

ref|XP_021537629.1| MSRDIYSTDYYRVGGRTMLPIRWMPPESILYRKFTTESDVWSFGVVLWEIFTYGKQPWYQ 730

ref|XP_035974161.1| MSRDIYSTDYYRVGGRTMLPIRWMPPESILYRKFTTESDVWSFGVVLWEIFTYGKQPWYQ 730

ref|XP_017495277.1| MSRDIYSTDYYRVGGRTMLPIRWMPPESILYRKFTTESDVWSFGVVLWEIFTYGKQPWYQ 730

ref|XP_036778484.1| MSRDIYSTDYYRVGGRTMLPIRWMPPESILYRKFTTESDVWSFGVVLWEIFTYGKQPWYQ 756

ref|XP_039107243.1| MSRDIYSTDYYRVGGRTMLPIRWMPPESILYRKFTTESDVWSFGVVLWEIFTYGKQPWYQ 730

ref|XP_019286763.1| MSRDIYSTDYYRVGGRTMLPIRWMPPESILYRKFTTESDVWSFGVVLWEIFTYGKQPWYQ 681

ref|XP_040308281.1| MSRDIYSTDYYRVGGRTMLPIRWMPPESILYRKFTTESDVWSFGVVLWEIFTYGKQPWYQ 740

ref|XP_023103311.1| MSRDIYSTDYYRVGGRTMLPIRWMPPESILYRKFTTESDVWSFGVVLWEIFTYGKQPWYQ 729

ref|XP_030158990.1| MSRDIYSTDYYRVGGRTMLPIRWMPPESILYRKFTTESDVWSFGVVLWEIFTYGKQPWYQ 729

ref|XP_026904149.1| MSRDIYSTDYYRVGGRTMLPIRWMPPESILYRKFTTESDVWSFGVVLWEIFTYGKQPWYQ 729

ref|XP_012514158.1| MSRDIYSTDYYRVGGRTMLPIRWMPPESILYRKFTTESDVWSFGVVLWEIFTYGKQPWYQ 730

ref|XP_012623143.1| MSRDIYSTDYYRVGGRTMLPIRWMPPESILYRKFTTESDVWSFGVVLWEIFTYGKQPWYQ 757

ref|XP_035135103.1| MSRDIYSTDYYRVGGRTMLPIRWMPPESILYRKFTTESDVWSFGVVLWEIFTYGKQPWYQ 730

ref|XP_003937906.1| MSRDIYSTDYYRVGGRTMLPIRWMPPESILYRKFTTESDVWSFGVVLWEIFTYGKQPWYQ 750

ref|XP_017365534.1| MSRDIYSTDYYRVGGRTMLPIRWMPPESILYRKFTTESDVWSFGVVLWEIFTYGKQPWYQ 756

ref|XP_032116570.1| MSRDIYSTDYYRVGGRTMLPIRWMPPESILYRKFTTESDVWSFGVVLWEIFTYGKQPWYQ 756

ref|XP_012305210.1| MSRDIYSTDYYRVGGRTMLPIRWMPPESILYRKFTTESDVWSFGVVLWEIFTYGKQPWYQ 750

ref|XP_023069875.1| MSRDIYSTDYYRVGGRTMLPIRWMPPESILYRKFTTESDVWSFGVVLWEIFTYGKQPWYQ 750

ref|XP_011783844.1| MSRDIYSTDYYRVGGRTMLPIRWMPPESILYRKFTTESDVWSFGVVLWEIFTYGKQPWYQ 756

ref|XP_033056540.1| MSRDIYSTDYYRVGGRTMLPIRWMPPESILYRKFTTESDVWSFGVVLWEIFTYGKQPWYQ 750

ref|XP_010379586.1| MSRDIYSTDYYRVGGRTMLPIRWMPPESILYRKFTTESDVWSFGVVLWEIFTYGKQPWYQ 750

ref|XP_017707989.1| MSRDIYSTDYYRVGGRTMLPIRWMPPESILYRKFTTESDVWSFGVVLWEIFTYGKQPWYQ 750

ref|XP_011848210.1| MSRDIYSTDYYRVGGRTMLPIRWMPPESILYRKFTTESDVWSFGVVLWEIFTYGKQPWYQ 756

ref|XP_007974896.1| MSRDIYSTDYYRVGGRTMLPIRWMPPESILYRKFTTESDVWSFGVVLWEIFTYGKQPWYQ 756

ref|XP_011934161.1| MSRDIYSTDYYRVGGRTMLPIRWMPPESILYRKFTTESDVWSFGVVLWEIFTYGKQPWYQ 756

ref|XP_003892892.4| MSRDIYSTDYYRVGGRTMLPIRWMPPESILYRKFTTESDVWSFGVVLWEIFTYGKQPWYQ 756

ref|XP_025213794.1| MSRDIYSTDYYRVGGRTMLPIRWMPPESILYRKFTTESDVWSFGVVLWEIFTYGKQPWYQ 756

ref|XP_011768197.2| MSRDIYSTDYYRVGGRTMLPIRWMPPESILYRKFTTESDVWSFGVVLWEIFTYGKQPWYQ 756

ref|XP_001114243.4| MSRDIYSTDYYRVGGRTMLPIRWMPPESILYRKFTTESDVWSFGVVLWEIFTYGKQPWYQ 756

ref|XP_005541454.2| MSRDIYSTDYYRVGGRTMLPIRWMPPESILYRKFTTESDVWSFGVVLWEIFTYGKQPWYQ 756

ref|XP_030679502.1| MSRDIYSTDYYRVGGRTMLPIRWMPPESILYRKFTTESDVWSFGVVLWEIFTYGKQPWYQ 721

ref|XP_032007428.1| MSRDIYSTDYYRVGGRTMLPIRWMPPESILYRKFTTESDVWSFGVVLWEIFTYGKQPWYQ 780

ref|XP_002810051.2| MSRDIYSTDYYRVGGRTMLPIRWMPPESILYRKFTTESDVWSFGVVLWEIFTYGKQPWYQ 750

ref|XP_004027043.1| MSRDIYSTDYYRVGGRTMLPIRWMPPESILYRKFTTESDVWSFGVVLWEIFTYGKQPWYQ 750

ref|**NP_002520.2| MSRDIYSTDYYRVGGRTMLPIRWMPPESILYRKFTTESDVWSFGVVLWEIFTYGKQPWYQ** 730

ref|XP_003308563.1| MSRDIYSTDYYRVGGRTMLPIRWMPPESILYRKFTTESDVWSFGVVLWEIFTYGKQPWYQ 756

ref|XP_004436300.1| MSRDIYSTDYYRVGGRTMLPIRWMPPESILYRKFTTESDVWSFGVVLWEIFTYGKQPWYQ 730

ref|XP_023496742.1| MSRDIYSTDYYRVGGRTMLPIRWMPPESILYRKFTTESDVWSFGVVLWEIFTYGKQPWYQ 730

ref|XP_014692423.1| MSRDIYSTDYYRVGGRTMLPIRWMPPESILYRKFTTESDVWSFGVVLWEIFTYGKQPWYQ 704

ref|XP_037681621.1| MSRDIYSTDYYRVGGRTMLPIRWMPPESILYRKFTTESDVWSFGVVLWEIFTYGKQPWYQ 731

ref|XP_020766508.1| MSRDIYSTDYYRVGGRTMLPIRWMPPESILYRKFTTESDVWSFGVVLWEIFTYGKQPWYQ 749

ref|XP_006052008.2| MSRDIYSTDYYRVGGRTMLPIRWMPPESILYRKFTTESDVWSFGVVLWEIFTYGKQPWYQ 729

ref|XP_019813710.1| MSRDIYSTDYYRVGGRTMLPIRWMPPESILYRKFTTESDVWSFGVVLWEIFTYGKQPWYQ 749

ref|XP_005898813.1| MSRDIYSTDYYRVGGRTMLPIRWMPPESILYRKFTTESDVWSFGVVLWEIFTYGKQPWYQ 733

ref|XP_002686012.2| MSRDIYSTDYYRVGGRTMLPIRWMPPESILYRKFTTESDVWSFGVVLWEIFTYGKQPWYQ 729

ref|XP_027385623.1| MSRDIYSTDYYRVGGRTMLPIRWMPPESILYRKFTTESDVWSFGVVLWEIFTYGKQPWYQ 729

ref|XP_010836511.1| MSRDIYSTDYYRVGGRTMLPIRWMPPESILYRKFTTESDVWSFGVVLWEIFTYGKQPWYQ 729

ref|XP_040086162.1| MSRDIYSTDYYRVGGRTMLPIRWMPPESILYRKFTTESDVWSFGVVLWEIFTYGKQPWYQ 729

ref|XP_027832376.1| MSRDIYSTDYYRVGGRTMLPIRWMPPESILYRKFTTESDVWSFGVVLWEIFTYGKQPWYQ 729

ref|XP_017901801.1| MSRDIYSTDYYRVGGRTMLPIRWMPPESILYRKFTTESDVWSFGVVLWEIFTYGKQPWYQ 749

ref|XP_007457931.1| MSRDIYSTDYYRVGGRTMLPIRWMPPESILYRKFTTESDVWSFGVVLWEIFTYGKQPWYQ 729

ref|XP_030697922.1| MSRDIYSTDYYRVGGRTMLPIRWMPPESILYRKFTTESDVWSFGVVLWEIFTYGKQPWYQ 755

ref|XP_026951067.1| MSRDIYSTDYYRVGGRTMLPIRWMPPESILYRKFTTESDVWSFGVVLWEIFTYGKQPWYQ 755

ref|XP_033712921.1| MSRDIYSTDYYRVGGRTMLPIRWMPPESILYRKFTTESDVWSFGVVLWEIFTYGKQPWYQ 755

ref|XP_004284580.2| MSRDIYSTDYYRVGGRTMLPIRWMPPESILYRKFTTESDVWSFGVVLWEIFTYGKQPWYQ 755

ref|XP_022415255.1| MSRDIYSTDYYRVGGRTMLPIRWMPPESILYRKFTTESDVWSFGVVLWEIFTYGKQPWYQ 729

ref|XP_024603973.1| MSRDIYSTDYYRVGGRTMLPIRWMPPESILYRKFTTESDVWSFGVVLWEIFTYGKQPWYQ 729

ref|XP_032493558.1| MSRDIYSTDYYRVGGRTMLPIRWMPPESILYRKFTTESDVWSFGVVLWEIFTYGKQPWYQ 729

ref|XP_029086756.1| MSRDIYSTDYYRVGGRTMLPIRWMPPESILYRKFTTESDVWSFGVVLWEIFTYGKQPWYQ 544

ref|XP_007110395.1| MSRDIYSTDYYRVGGRTMLPIRWMPPESILYRKFTTESDVWSFGVVLWEIFTYGKQPWYQ 729

ref|XP_036692187.1| MSRDIYSTDYYRVGGRTMLPIRWMPPESILYRKFTTESDVWSFGVVLWEIFTYGKQPWYQ 729

ref|XP_010952367.1| MSRDIYSTDYYRVGGRTMLPIRWMPPESILYRKFTTESDVWSFGVVLWEIFTYGKQPWYQ 691

ref|XP_015103644.1| MSRDIYSTDYYRVGGRTMLPIRWMPPESILYRKFTTESDVWSFGVVLWEIFTYGKQPWYQ 661

ref|XP_032319890.1| MSRDIYSTDYYRVGGRTMLPIRWMPPESILYRKFTTESDVWSFGVVLWEIFTYGKQPWYQ 729

ref|XP_031291913.1| MSRDIYSTDYYRVGGRTMLPIRWMPPESILYRKFTTESDVWSFGVVLWEIFTYGKQPWYQ 729

****:******** *********:*****::  ****:*****: *:*******

**B. PSKR1 orthologues**

****:*:**:**:* :****:: : . :::: :. : : :.. :

XP_022989696.1 YSFGVVLLELLTGKRPIDMCRPKGVRDLISWVFQIREDRKVSEVFDPLVYNKQHE-TAMT 972

KAG7030528.1 YSFGVVLLELLTGKRPIDMCRPKGVRDLISWVFQIREDRKVSEVFDPLIYNKQHE-TAMS 972

XP_023517407.1 YSFGVVLLELLTGKRPIDMCRPKGVRDLISWVFQIREDRKVSEVFDPLIYNKQHE-TAMS 972

XP_022942343.1 YSFGVVLLELLTGQRPIDMCRSKGLQDLVSWVFEMRNDRKISEVFDPLIYDKKNE-RAMA 974

KAA0035969.1 YSFGVVLLELLTGKRPIDMCRPKGLRDLISWVFQMRKDKKVSEVFDTLVYDKKNE-TVMV 974

XP_008460236.1 YSFGVVLLELLTGKRPIDMCRPKGLRDLISWVFQMRKDKKVSEVFDTLVYDKKNE-TVMV 974

XP_023766126.1 YSFGVVLLELLTGKRPMDMCKPKGSRDLISFVMQMKREKRENEVFDSFVFDKDNA-KGML 982

GEX26021.1 YSFGVVLLELLTGKRPMDMCKPRGSRDLISWVKQMKMEKRETEVIDSFIFNKEHS-NEML 971

XP_022025970.1 YSFGVVLLELLTGRRPMDMCKPKGGRELISWVMQMKTEKRENEVMDSCIFNKEQA-SEML 984

XP_043638587.1 YSFGVVLLELLTGKRPMDMCKPKGSRDLISWVMQMKIEKRETEVIDSLIFDKDRA-KEML 981

XP_030447632.1 YSFGVVLLELVTGKRPMDMCKPKGCRDLISWVIKMKSETRESEVFDRSIYGTKHD-KEML 987

XP_031374250.1 YSFGVVLLELLTGKRPMDICKPKGSRDLISWVIQMKRDGREGEVFDQNIYGKQHD-KEIL 989

KAF5176079.1 YSFGVVLLELLTGKRPMDMCKPKDRRNLISWVFQMKKEKKEAEVIDPFIYDKQHN-KEIL 938

CAA2959270.1 YSFGVVLLELLTGKRPMDMCKPKSRRDLISWVIQMKSEKRETEVFDPFIYNKENA-DEML 990

XP_022890808.1 YSFGVVLLELLTGKRPMDMCKSKSRRDLISWVIQMKSEKRETEVFDPFIYNKENA-DEML 990

KZV52175.1 YSFGVVLLELLTSKRPMDMCRPKEYRDLISWVIQMKREKRETEVFDPFIYDKQYT-EEML 986

XP_011070486.1 YSFGVVLLELLTSKRPMDMCRPRASRDLISWVAQMKREKRETEVFDPFIYDKQHA-PQML 981

KAH6792628.1 YSFGVVLLELLTGKRPMDIGRPKGCRDMIAWVIQMKREKRETEVVDPFIYDKQHA-QEML 976

XP_027100174.1 YSFGVVLLELLTGKRPMDMCKPKENRDLISWVIQQKKDKRETEVFDPFIYEKEHA-EELL 978

XP_027150704.1 YSFGVVLLELLTGKRPMDMCKPKENRDLISWVIQQKKDKRETEVFDPFIYEKEHA-EELL 978

XP_031112941.1 YSFGVVLLELLTAKRPMDMCKPKGSRDLISWVIQMKKEKRQTEVFDPLVYDKQHA-DEML 992

XP_009604086.1 YSFGVVLLELLTGKRPMDPCKPRASRDLISWVKQLKKQKRETEVFDPLIYDKQHA-EEML 986

XP_019228282.1 YSFGVVLLELLTGKRPMDPCKPRASRDLISWVIQLKKQKRETEVFDPLIYDKQHA-QEML 972

XP_009782544.1 YSFGVVLLELLTGKRPMDPCKPRASRDLISWVIQLKKQKRETEVFDPLVYDKQHA-QEML 987

KAF3678827.1 YSFGVVLLELLTCKRPMDPCKPRANRNLISWVIQMKQQKRETEVFDPLIYDKQHA-QEML 975

PHT60396.1 YSFGVVLLELLTCKRPMDPCKPRANRNLISWVIQMKQQKRETEVFDPLIYDKQHA-QEML 986

XP_006348541.1 YSFGVVLLELLTCKRPMDPCKPRASRDLISWVIQMKKQKRETEVFDPLIYDKQHA-KEML 974

XP_015062793.1 YSFGVVLLELLTCKRPMDPCKPRASRDLISWVIQMKKQKRETEVFDPLIYDKQHA-KEML 974

XP_004228537.1 YSFGVVLLELLTCKRPMDPCKPRASRDLISWVIQMKKQKRETEVFDPLIYDKQHA-KEML 974

XP_028084276.1 YSFGVVLLELLTGKRPMDICKPKGSRNLISWVFQMKREKRESEVFDAFIYDKDHG-KEML 997

PSS26842.1 YSFGVVLLELLTGKRPMDMCKAKENRDLISWVLRMRREKLETQVFDPFIYDKQKS-KEML 986

XP_010527857.1 YSFGVVLLELVTGKRPMDMCRPKGSRDLISWVVKMKNENRASEVFDPFIYDKDHD-KQML 988

XP_006395801.1 YSFGVVLLELLTDKRPVDMCKPKGGRDLISWVVRMKNENRASEVFDPLIHGKENE-KEML 986

**NP_178330.1 YSFGVVLLELLTDKRPVDMCKPKGCRDLISWVVKMKHESRASEVFDPLIYSKEND-KEMF 978**

KAG7640263.1 YSFGVVLLELLTDKRPVDMCKPKGCRDLISWVVKMKHESRASEVFDPLIYSKEND-KEMF 978

EFH53063.1 YSFGVVLLELLTDKRPVDMCKPKGCRDLISWVVKMKHENRASEVFDPLIYSKEND-KEMF 978

XP_010424999.1 YSFGVVLLELLTDKRPVDMCKPKGCRDLISWVVKMKHENRASEVFDPLVYSREKD-KEMC 986

XP_006292801.1 YSFGVVLLELLTDKRPVDMCKPKGSRDLISWVVKMKYENRASEVFDPLIYRKENE-KEML 986

XP_009129163.1 YSFGVVLLELLTDRRPVDMCKPKGGRDLISWVVRMKSEGRASEVFDPFIHGKENE-KEMV 913

XP_018484363.1 YSFGVVLLELLTDKRPVDMCKPKGGRDLISWVVRMKSEGRASEVFDPFIHGKEIEEEEMF 989

XP_013598267.1 YSFGVVLLELLTDKRPVDMCKPKGGRDLISWVVRMKSEGRASEVFDPFIHGKV-NEEEMF 974

XP_013707217.1 YSFGVVLLELLTDKRPVDMCKPKGGRDLISWVVRMKSEGRASEVFDPFIHGKV-NEEEMF 974

XP_042511280.1 YSFGVVLLELLTGKRPVDMCKPEGCRDLISWVLRMKKEKRESEVFDPLIYGKQND-KELL 985

XP_043695420.1 YSFGVVLLELLTGKRPVDMCKPEGCRDLISWVLQMKKEKRESEVFDPFIHDKQND-KEML 985

XP_044490312.1 YSFGVVLLELLTGKRPMDMCKPRGCRNLISWVIQMKEENRESEVLDPFIYDKKHD-KELL 1039

XP_024035321.1 YSFGVVLLELLTGKRPMDMCKPKGSRDLISWVIRMRQENRESEVLDPFIYDKQHD-KEML 989

XP_024952188.1 YSFGVVLLELLTGKRPMDMCKPKGSRDLISWVIRMRQENRESEVLDPFIYDKQHD-KEML 989

KAE8663891.1 YSFGVVLLELMTGKRPMDMCKPKGSRDLISWVIHMKMENKETEVFDPYIHDKQHE-KETL 914

XP_017632150.1 YSFGVVLLELLTGKRPMDMCKPKGTRDLISWVIRMKMENKESEVFDPFIYGKQHD-KEML 976

XP_012478870.1 YSFGVVLLELLTGKRPMDMCKPKGTRDLISWVIRMKMENKESEVFDPFIYGKQHD-KEML 976

XP_022776608.1 YSFGVVLLELLTAKRPMDMCKPKGSRDLISWVIRMKMENKESEVFDSFIYEKQHD-EEML 978

XP_021293147.1 YSLGVVLLELLTGKRPMDMCKPKGSRDLISWVIRMKIENKESEVFDPFIYGKQHD-KEML 978

XP_017971937.1 YSFGVVLLELLTGKRPMDMCKPRGSRNLISWVIRMKIENRESEVFDPFIYGKQHD-KEML 978

XP_038694271.1 YSFGVVLLELLTGKRPMDMCKPRGSRDLISWAIQMKKDHRENEVFDPFIYGKQHE-KEMI 982

KAG5240598.1 YSFGVVLLELLTGKRPMDMCKPKGSRDLISWVIQMKKENRESEVFDPFIYDKQND-KELH 964

XP_034922712.1 YSFGVVLLELLTGKRPMDMCKPKGSRDLISWVIEMKKENRESEVFDPFIYDKQND-KELQ 991

XP_002312507.2 YSFGVVLLELLTGKRPMDMCKPKGSRDLISWVIQMKKENRESEVFDPFIYDKQND-KELQ 991

XP_011025322.1 YSFGVVLLELLTGKRPMDMCKPKGSRDLISWVIEMKKENRESEVFDPFIYDKQND-KELQ 991

XP_021618701.2 YSFGVVLLELLTGKRPMDMCKPKGSRDLISWVIQMKKENRESEVFDPFICDKQHD-KQLL 1001

XP_021672714.1 YSFGVVLLELLTGKRPMDMCKPKGSRDLISWVIQMKKENRESEVFDPFIYDKQND-KQLL 991

XP_002518809.1 YSFGVVLLELLTGKRPMDMCKPKGSRDLISWVIQMKKENRESEVFDPFIYDKQND-KQLL 976

XP_012081968.1 YSFGVVLLELLTGKRPMDMCKPKGSRDLISWVLQMKKENRESEVFDPFIYGKDND-KQLL 984

XP_030507333.1 YSFGVVLLELLTGKRPMDMCKPKGCRDLISWVFQMKKEKKESEVFDPFIWDKDHN-KELL 995

XP_010092928.1 YSFGVVLLELLTGKRPMDMCKPKGCRDLISWVFQMKKEKKESEVFDPFIYNKHND-KELL 974

XP_015895857.1 YSFGVVLLELLTGKRPMDMCKPKGSRDLISWVFQMKREKRECEVFDPFIYDKQHN-EELM 986

XP_024193533.1 YSFGVVLLELLTGKRPMDMCKPKGARDLISWVFQMKRERRETEVFDPVIYDKQRE-QELL 986

XP_004297774.1 YSFGVVLLELLTGKRPMDMCKPKVARDLISWVFQMKREKRETEVFDPVIYDKQKD-QELL 987

XP_008221004.1 YSFGVVLLELLTGKRPMDMCKPRGCRDLISWAFQMKREKRETEVFDPFIYDKKHD-EELL 986

XP_034213209.1 YSFGVVLLELLTGKRPMDMCKPRGCRDLISWAFQMKREKRETEVFDPFIYDKKHD-EELL 987

XP_007227028.1 YSFGVVLLELLTGKRPMDMCKPRGCRDLISWAFQMKREKRETEVFDPFIYDKKHD-EELL 987

PQP95369.1 YSFGVVLLELLTGKRPMDMCKPRGCRDLISWVFQMKKEKRETEVFDPFIYDKKHD-EELL 987

XP_021811392.1 YSFGVVLLELLTGKRPMDMCKPRGCRDLISWAFQMKREKRETEVFDPFIYDKKHD-EELL 987

XP_008340497.1 YSFGVVLLELLTGKRPMDMCKPKECRDLISWAFQMKREKKESEVFDPFICDKQHD-EELL 986

KAB2624072.1 YSFGVVLLELLTGKRPMDMCKPKECRDLISWAFQMKREKRESEVFDPFICDKQHD-EELL 986

XP_009351664.1 YSFGVVLLELLTRKRPMDMCKPKECRDLISWAFQMKREKRESEVFDPFICDKQHD-EELL 986

XP_002273186.2 YSFGVVLLELLTGKRPMDMCKPRGCRDLISWVIQMKKEKRESEVFDPFIYDKQHD-KELL 989

XP_034692513.1 YSFGVVLLELLTGKRPMDMCKPRGCRDLISWVIQMKKEKRESEVFDPFIYDKQHD-KELL 989

XP_030959993.1 YSFGVVLLELLTGKRPMDMCKPKGFRDLISWVFEMKKENRESEVFDPFVYDKQND-KEML 986

XP_023879078.1 YSFGVVLLELLTGKRPMDMCKPKGSRDLISWVFEMKKENRESEVFDPFVYDKQND-KEML 986

KAB1206051.1 YSFGVVLLELLTGKRPMDMCKPKGSRDLISWVFQMKNENRTSEVFDPFIYDKQHD-QEML 984

XP_041012192.1 YSFGVVLLELLTGKRPMDMCKPKGSQDLISWVFQMKKENRQNEVFDPFIYDKQHD-REML 983

XP_042980779.1 YSFGVVLLELLTGKRPMDMCKPKGSQDLISWVFQMKKENRQNEVFDPLIYDKQHD-REML 974

KAG2371969.1 YSFGVVLLELLTARRPVEVIKGKNCRNLVSWVFQMKSENKEQEIFDPAIWHKDHE-KQLL 1017

XP_015636832.1 YSFGIVLLELLTGRRPVDMCRPKGSRDVVSWVLQMKKEDRETEVFDPTIYDKENE-SQLI 979

KAE8773508.1 YSFGIVLLELLTGRRPVDMCRPKGSRDVVSWVLQMRKEDRETEVFHPNVHDKANE-GELL 1003

XP_020581339.1 YSFGVVLLELLTGRRPVDITKPKGSRELVLWVLKMK-ERKETELFDPIIYRKELE-KQLL 1003

RWR92883.1 YSFGVVLLELLTGKRPVEVCKPKGCRDLIAWVLQMKKEKRENEVFDPFLCDKQCN-KQML 1003

PWZ22553.1 YSFGIVLLELLTGKRPIDMCKPKGARELVSWVTLMKKENREADVLDRAMYDKKFE-TQMR 1036

XP_004953174.1 YSFGIVLLELLTGKRPVDMCKPKGARELVSWVTHMKKENRETDVLDRAMYDKKFE-KEMM 1013

XP_002454207.1 YSFGIVLLELLTGKRPVDMCKPKGARELVSWVTHMKKENREADVLDRAMYDKKFE-TQMI 1016

EMS56714.1 YSFGIVLLELLTGKRPVDMCKRKGARELVSWVMHMKGEHREADVLDRAMYDKKFE-MQMM 1014

XP_014755449.1 YSFGIVLLELLTGKRPVDMCKPKGARELVSWVIHMKGENREADVLDRAMYEKKYE-IQMM 1087

PKA52141.1 YSFGVVLLELLTGKRPIDMTKPKSGRDLISWVLQMRKERREYEVFDSFIYSKEHE-DQMT 1002

XP_020674731.1 YSFGVVLLELLTGKRPVDMSKPKSCRDLISWVLQMRKEGMEAYVLDRIIYNKENE-GQMV 1002

OAY82638.1 YSFGVVLLELLTGKRPVDMCKPKGGRELISWVLQMKKENHVAEVFDPLIYEKKHT-NQLM 1018

EHA8589572.1 YSFGVVLVELLTGKRPVDMCRPKGGRDLISWVLQMKKEKRENEVFDPFIYDKKHD-SQMK 799

****:*:**:**:* :****:: : . :::: :. : : :.. :

**Figure S2**. Alignment of the guanylate cyclase (GC) center of NTRK1 (A) and PSKR1 (B).

The sequence logos in Figure 1 were generated from the alignments presented here. Human NTRK1 sequence (NP_002520.2) is marked in bold brown text and Arabidopsis PSKR1 sequence (NP_178330.1) is marked in bold green text. Alignment generated by Clustal Omega from 217 NTRK1 orthologs and 98 PSKR1 orthologs. The GC centre is marked where identical residues are indicated with an asterisk (*), conserved residues with a colon (:), and semi-conserved residues with a full stop (.) in magenta.
